# The AraC Negative Regulator family modulates the activity of histone-like proteins in pathogenic bacteria

**DOI:** 10.1371/journal.ppat.1006545

**Published:** 2017-08-14

**Authors:** Araceli E. Santiago, Michael B. Yan, Tracy H. Hazen, Brooke Sauder, Mario Meza-Segura, David A. Rasko, Melissa M. Kendall, Fernando Ruiz-Perez, James P. Nataro

**Affiliations:** 1 Department of Pediatrics, University of Virginia School of Medicine, Charlottesville, Virginia, United States of America; 2 Institute for Genome Sciences, Department of Microbiology and Immunology. University of Maryland, Baltimore, Maryland, United States of America; 3 Department of Microbiology, Immunology, and Cancer Biology, University of Virginia, Charlottesville, Virginia, United States of America; Universite Paris Descartes, FRANCE

## Abstract

The AraC Negative Regulators (ANR) comprise a large family of virulence regulators distributed among diverse clinically important Gram-negative pathogens, including *Vibrio* spp., *Salmonella* spp., *Shigella* spp., *Yersinia* spp., *Citrobacter* spp., and pathogenic *E*. *coli* strains. We have previously reported broad effects of the ANR members on regulators of the AraC/XylS family. Here, we interrogate possible broader effects of the ANR members on the bacterial transcriptome. Our studies focused on Aar (AggR-activated regulator), an ANR family archetype in enteroaggregative *E*. *coli* (EAEC) isolate 042. Transcriptome analysis of EAEC strain 042, 042*aar* and 042*aar*(pAar) identified more than 200 genes that were differentially expressed (+/- 1.5 fold, p<0.05). Most of those genes are located on the bacterial chromosome (195 genes, 92.85%), and are associated with regulation, transport, metabolism, and pathogenesis, based on the predicted annotation; a considerable number of Aar-regulated genes encoded for hypothetical proteins (46 genes, 21.9%) and regulatory proteins (25, 11.9%). Notably, the transcriptional expression of three histone-like regulators, H-NS (*orf1292*), H-NS homolog (*orf2834*) and StpA, was down-regulated in the absence of *aar* and may explain some of the effects of Aar on gene expression. By employing a bacterial two-hybrid system, LacZ reporter assays, pull-down and electrophoretic mobility shift assay (EMSA) analysis, we demonstrated that Aar binds directly to H-NS and modulates H-NS-induced gene silencing. Importantly, Aar was highly expressed in the mouse intestinal tract and was found to be necessary for maximal H-NS expression. In conclusion, this work further extends our knowledge of genes under the control of Aar and its biological relevance *in vivo*.

## Author summary

The AraC Negative Regulators (ANR) is a large family of negative regulators distributed in several clinically relevant Gram-negative pathogens, including *Vibrio* spp., *Salmonella* spp., *Shigella* spp., *Yersinia* spp., *Citrobacter* spp., pathogenic *E*. *coli*, and members of the *Pasteurellaceae*. Previously, we showed that the ANR family suppresses transcriptional expression of virulence factors such as fimbriae, toxins, and the type VI secretion system by directly down-regulating AraC/XylS master regulators. Transcriptome and biochemical analysis of Aar (AggR-activated regulator), an ANR family archetype in enteroaggregative *E*. *coli* (EAEC) 042, demonstrated that Aar binds directly to H-NS and modulates the H-NS-induced gene expression. Accordingly, mutation of *aar* decreased expression of the H-NS-regulated Lpf fimbriae, LPS-related enzymes, GadXW operon and porins. Importantly, Aar was highly expressed in the mouse intestinal tract and was found to be necessary for maximal H-NS expression. These findings unveil an exquisite regulatory network in pathogenic bacteria, which operates by concomitant control of master transcriptional regulators of the AraC family and global negative H-NS regulators.

## Introduction

Pathogenic bacteria utilize elaborate regulatory mechanisms to effect appropriate expression of virulence-associated traits. The availability of genomic data sets and new high-throughput methods have illuminated not only a large number of new virulence loci, but also exposed previously unappreciated regulatory systems. We have recently described the ANR (AraC Negative Regulators) family, a large family of bacterial gene regulators expressed by diverse clinically important Gram-negative pathogens. Organisms implicated include *Vibrio* spp., *Salmonella* spp., *Shigella* spp., *Yersinia* spp., *Citrobacter* spp., pathogenic *E*. *coli* strains including enterotoxigenic (ETEC) and enteroaggregative *E*. *coli* (EAEC), and members of the *Pasteurellaceae* [1,2]. Genes coding for ANR are variously present either on the chromosome or on plasmids [1].

EAEC is a diarrheagenic pathotype linked to traveler’s diarrhea, foodborne outbreaks and sporadic diarrhea in industrialized and developing countries [3–8]. The ability of this pathogen to colonize the mucosa is attributed to the presence of virulence factors regulated by the transcriptional activator AggR [9–14]. Aar (AggR activated regulator) was the first characterized ANR protein, identified in EAEC strain 042 by its ability to repress AggR activity [1,2,13].

Aar is a 63-amino-acid protein with a molecular mass of 7.23 kDa. The protein comprises three alpha helical domains required for oligomerization [2]. Aar does not have apparent DNA binding capability, but instead binds to the AggR protein directly, thus inhibiting the latter’s ability to bind to bacterial promoter regions [2]. Disruption of *aar* leads to increased expression of AggR and its regulon, which comprises at least 44 genes with putative virulence functions [1,13]. ANR homologs of *Vibrio cholerae*, *Citrobacter rodentium*, *Salmonella enterica* and ETEC rescued *aar* mutants in EAEC strain 042 [1,2].

H-NS (Histone-like Nucleoid Structuring) is a nucleoid-associated protein widely distributed among Gram-negative bacteria [15,16]. In *E*. *coli*, up to 5 to 10% of the genome is subject to H-NS-dependent regulation [17,18]. H-NS binds AT-rich regions thought to be acquired by horizontal gene transfer [19–22], and such regions are characteristic of many virulence-associated loci. Oligomerization of H-NS is critical for its regulatory activity [23–26]. The H-NS protein is not only capable of interacting directly with DNA but also with other regulatory proteins and itself [23,25,27]. One of the best-characterized H-NS binding partners is its paralog, StpA [28,29].

The main purpose of this study is to characterize the global regulatory effects of the ANR family member Aar. We selected EAEC as a model organism in which to study the biological relevance of the ANR family, and performed a global transcriptome analysis of EAEC strain 042, its mutant 042*aar* and complemented in trans strain 042*aar*(pAar); 210 genes were differentially expressed. Further functional studies suggested that Aar not only interacts with members of the AraC family [2], but also with members of the histone-like family (*orf1292*, *orf2834* and *stpA*). We assess here the effect of Aar-H-NS interaction in H-NS-mediated regulation.

## Results

### Global regulatory effects of Aar

**Fig 1:**
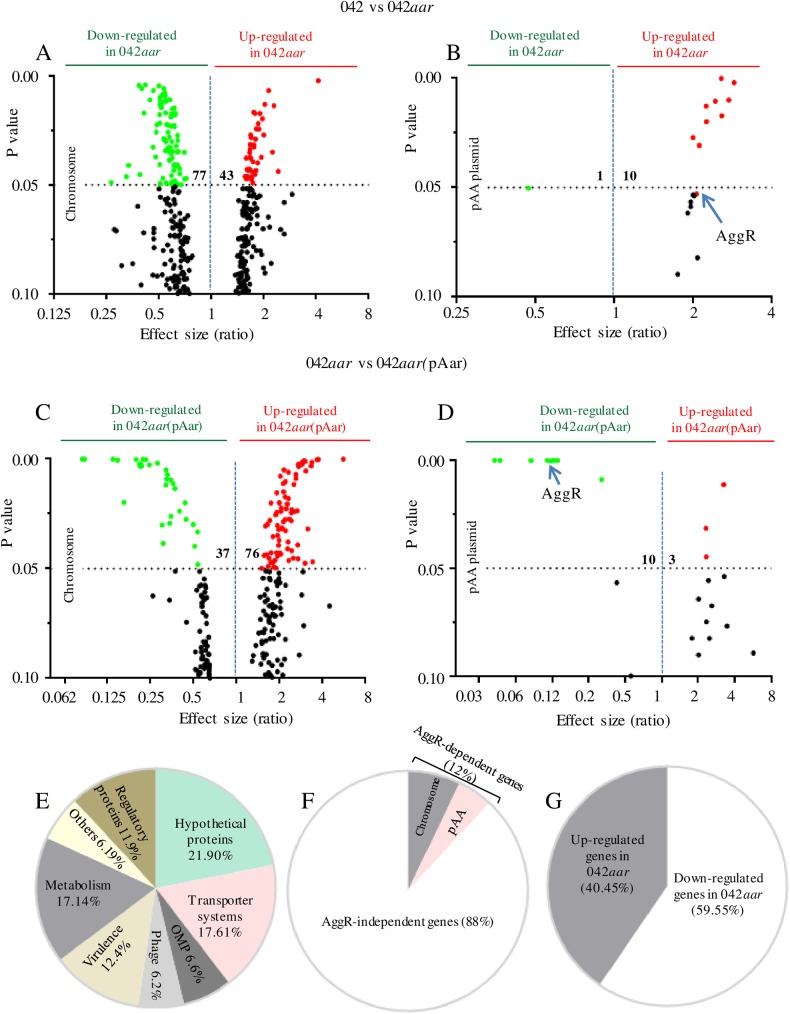
RNA-seq analysis of the Aar regulon. Comparison of differentially expressed genes for EAEC strain 042 vs. 042*aar* (panel A and B) and 042*aar* vs 042*aar*(pAar) (panel C and D) are showed in the volcano graphs. The genes were grouped in eight major categories based on the predicted NCBI annotation (panel E), AggR requirements (panel F) and up or down-regulated genes (panel G).

We evaluated the global effect of Aar in EAEC 042 by using RNA-seq technology. RNA was extracted from wild type (wt) 042, its isogenic 042*aar* mutant and complemented in trans 042*aar*(pAar) strain grown in DMEM-high glucose, which activates expression of the master transcriptional regulator AggR and AggR-dependent Aar [13]. The samples were converted into cDNA libraries using the Ovation Prokaryotic RNA-Seq System (NuGen) and sequenced on the Illumina HiSeq 2000 as indicated in material and methods. We identified 210 genes that were differentially expressed (+/- 1.5 fold, p<0.05) (Fig 1). Aar-regulated genes were mainly found on the chromosome (195 genes, 92.85%), and were grouped in eight major categories: hypothetical proteins (21.9%), proteins involved in metabolic functions (17.14%), transporter proteins (17.61%), regulatory proteins (11.9%), putative virulence factors (12.4%), membrane proteins (6.6%), phage proteins (6.2%), and proteins involved in diverse other functions (6.19%) (Fig 1E and S1 to S8 Figs). In the pAA plasmid, the Aar-regulated genes were found principally in AT-rich regions (S9 Fig). Putative gene assignments and homologies of the differentially transcribed genes evaluated in this study are listed in Table 1.

**Table 1:** Genes evaluated in this study.

ORF	Gene (NCBI)	Function
	**Hypothetical proteins**	
Orf1228	CBG34048.1	Uncharacterized conserved protein YcgL, UPF0745 family
Orf2823	CBG35656.1	Hypothetical protein. No putative conserved domains have been detected
Orf3192	CBG36017.1	Hypothetical protein, DUF3296 family
Orf3205	CBG36033.1	Conserved protein. No putative conserved domains have been detected
Orf3334	CBG36162.1	Conserved hypothetical protein, DUF4051 family
Orf4746	CBG37565.1	Conserved hypothetical protein, DUF4007 family
Orf4753	CBG37572.1	Conserved hypothetical protein, DUF4432 family
	**Transporter proteins**	
Orf0690	CBG33516.1	Glutamate/aspartate ABC transporter, substrate-binding protein
Orf4080	CBG36905.1	Phosphate ABC transporter, substrate-binding protein
	**Putative virulence factors**	
Orf3928	CBG36751.1	Putative lipopolysaccharide biosynthesis protein
Orf3931	CBG36754.1	Lipopolysaccharide heptosyltransferase I
Orf3932	CBG36755.1	Lipid A-core:surface polymer ligase
Orf4082	CBG36907.1	Fimbrial outer membrane usher protein, LpfC
	**Transcriptional factors**	
pAA052	CBG27804.1	AraC/XylS family transcriptional regulator AggR
Orf0808	CBG33632.1	Regulatory protein cro (antirepressor)
Orf1127	CBG33946.1	Biofilm regulator, BssS
Orf2020	CBG34846.1	Putative hex-regulon repressor (RpiR-family transcriptional regulator)
Orf2058	CBG34884.1	Transcriptional activator FlhD
Orf2881	CBG35713.1	MarR-family transcriptional repressor
Orf2888	CBG35720.1	Carbon storage regulator
Orf3045	CBG35872.1	Putative transcriptional regulator. Conserved protein domain family HTH
Orf3191	CBG36016.1	Putative DNA binding protein.
Orf3204	CBG36032.1	Putative regulatory protein
Orf4499	CBG37321.1	transcriptional activator, CadC
Orf4555	CBG37377.1	Putative transcriptional regulator
	**Outer membrane Protein**	
Orf0904	CBG33729.1	Outer membrane protein X (OmpX)
Orf1042	CBG33864.1	Outer membrane protein A (OmpA)
Orf1904	CBG34730.1	Osmotically inducible lipoprotein E (OsmE)
	**Global regulators**	
Orf1292	CBG34112.1	DNA-binding protein (histone-like protein Hlp-II), H-NS protein
Orf2834	CBG35667.1	Putative histone-like DNA-binding protein
StpA	CBG35697.1	DNA-binding protein, StpA protein
**Acid resistance operon**		
Orf3806	CBG36631.1	Transcriptional regulator GadE
Orf3810	CBG36635.1	AraC/XylS family transcriptional regulator GadW
Orf3811	CBG36636.1	AraC/XylS family transcriptional regulator GadX
	**Others**	
pAA055	CBG27807.1	Dispersin, Aap
Orf2223	CBG35049.1	AMP nucleosidase

Forty-six differentially-regulated genes were assigned as hypothetical proteins (S1 Fig). Seven out of forty-six hypothetical proteins were encoded on the pAA plasmid, including the AggR-regulated pAA047 gene. In agreement with previous findings, AggR-regulated genes (23 out of 44) were up-regulated in the absence of *aar* (depicted in yellow, S1, S2, S4, S5 and S8 Figs) [13]. Unexpectedly, we observed that the majority of genes regulated by Aar (88%) were not dependent on transcriptional activator AggR (Fig 1F).

**Fig 2:**
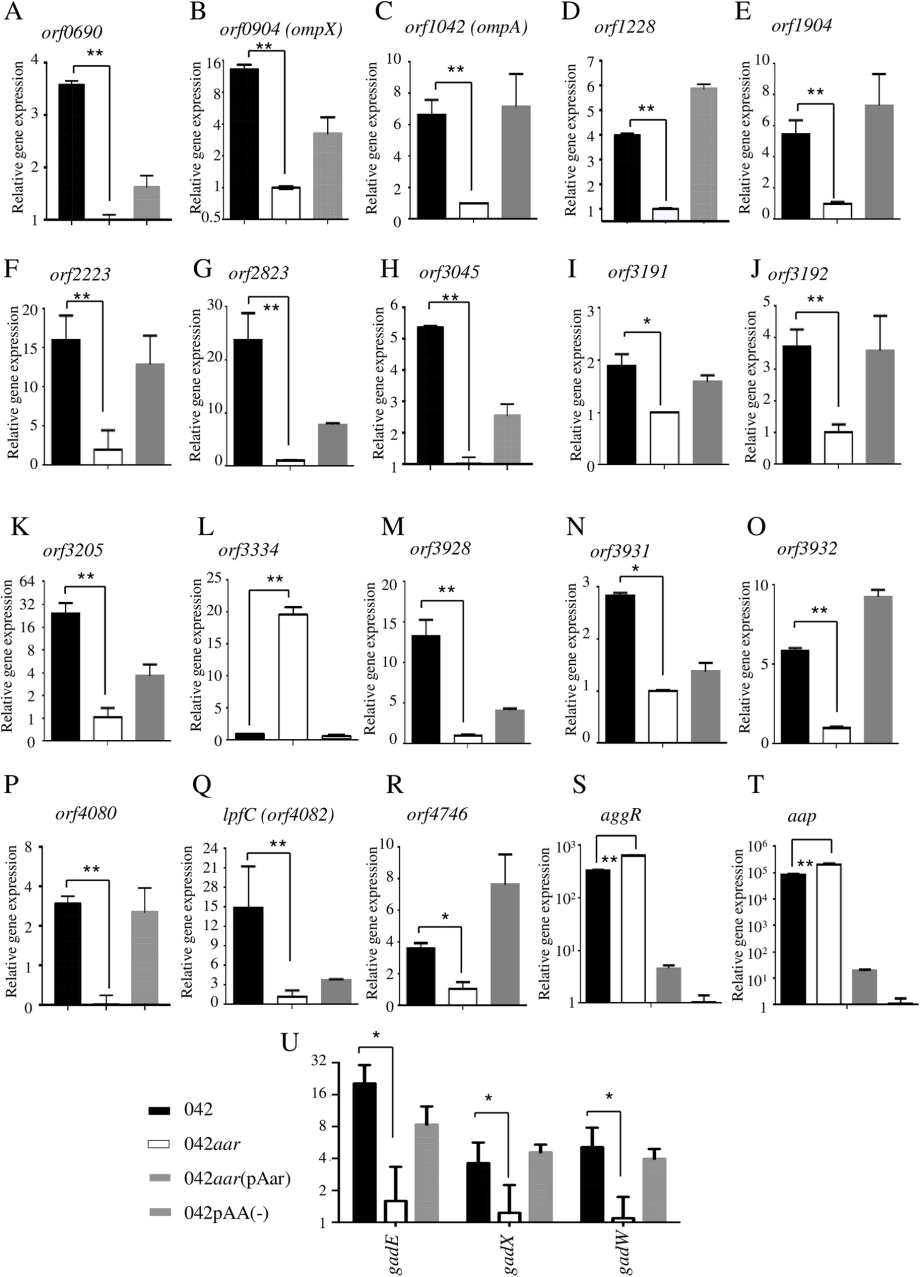
Validation of Aar-regulated genes by qRT-PCR. Transcriptional levels of Aar-regulated genes were quantitated by qRT-PCR in 042 (black bars), 042*aar* (open bars) and 042*aar*(pAar) (gray bars). Expression levels for each queried gene were normalized to the constitutively expressed *cat* gene in EAEC 042. Samples A to R, and U were compare against 042*aar* strain. Panels S and T were compare against 042 pAA(-). Data are representative of at least three independent experiments. Asterisks indicate significant difference by ANOVA (*, P < 0.01; **, P < 0.001).

We validated our transcriptome database by qRT-PCR. Twenty-three genes were selected for this analysis based on the p-value and consistency between the groups in the database, as well as potential biological relevance. These genes included hypothetical proteins *orf1228*, *orf2823*, *orf3192*, *orf3205*, *orf3334* and *orf4746*; transporter proteins *orf0690* and *orf4080*; putative virulence factors *orf3928*, *orf3931*, *orf3932*, *orf4082* (l*pfC*); transcriptional factors *aggR*, *orf3045*, *orf3191*, *gadEWX*; AggR-regulated dispersin (*aap*); outer membrane proteins *orf0904* (*ompX*), *orf1042* (*ompA*), *orf1904* (*osmE*) and hypothetical *orf2223* (Fig 2). Strains were grown in DMEM-high glucose for 5 h (late log phase) as previously standardized [1], and total RNA was isolated and prepared for qRT-PCR. Our data showed that 20 out of 23 analyzed genes exhibited lower expression in the 042*aar* mutant (~2 to 20 fold) (Fig 2). These findings were consistent with the RNA-seq data, which revealed that the majority of genes (78 genes out of 131) were down regulated in 042*aar* compared to wild type 042 (Fig 1A and 1B). Only 3 out of 23 genes (*orf3334*, *aggR*, and AggR-dependent *aap*) showed greater levels of expression in the 042*aar* strain (Fig 2L, 2S and 2T). Complementation *in trans* with the Aar gene resulted in wild type levels in most of the cases (Fig 2).

### Aar regulates members of the AraC/XylS family

We reported that Aar binds with high affinity to the central linker domain of AraC-like members and abolishes their DNA binding activity [2]. In agreement with this report, two members of the AraC family, AggR and GadX, were detected in the transcriptome database (S5 Fig). As expected, we observed that 042*aar* shows increased expression of *aggR* in late logarithmic growth phase (~2 fold) (Fig 2S). A similar effect was observed for AggR-regulated genes including *aap* (Fig 2T); these included pAA plasmid genes *aafA*, *aafB*, *aafD*, *aatA*, pAA003, pAA005, pAA005A, pAA047, and chromosomally-encoded genes *orf3182*, *orf3184*, *orf4562*, *orf4563*, *orf4564*, *orf4565*, *orf4568*, *orf4569*, *orf4570*, *orf4572*, *orf4574*, *orf4574A*, *orf4576*, *orf4581 and orf4582* (depicted in yellow, S1, S2, S4, S5 and S8 Figs). In contrast to *aggR*, transcription of *gadX* in 042 was reduced after mutation of *aar* (Fig 2U). We observed not only *gadX* but also its downstream gene *hdeB* to be regulated by Aar [2]. To confirm the regulatory effect of Aar on the acid resistance operon in 042, we evaluated the expression of the transcriptional *gadE* and *gadW* genes by qRT-PCR. We observed that the three transcriptional regulators GadEWX of the operon were down-regulated in 042*aar* (Fig 2U) suggesting that the acid resistance operon may be affected by Aar.

### Aar affects expression of histone-like proteins

**Fig 3:**
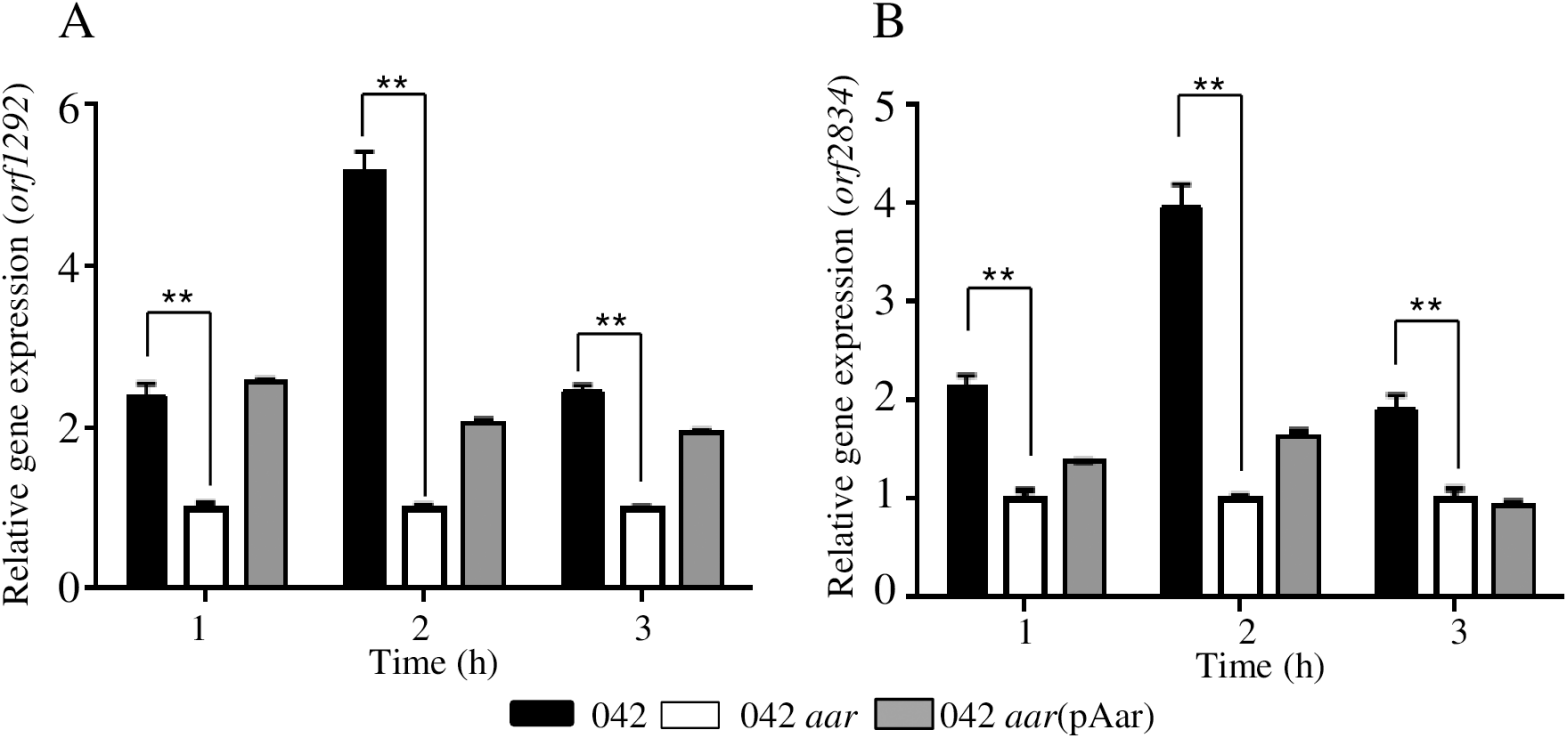
Histone-like proteins are regulated by Aar. Transcriptional levels of *orf1292* (A) and *orf2834* (B) were quantified by qRT-PCR in 042 (black bars), 042*aar* (open bars) and 042*aar*(pAar) (gray bars). Data are representative of at least three independent experiments. Asterisks indicate significant difference by ANOVA (**, P < 0.001).

Transcriptional levels of 25 transcriptional factors were affected by Aar in 042 (S5 Fig). However, only six out of the 25 transcriptional factors were complemented in trans by Aar, including AggR, H-NS (*orf1292*), putative H-NS homolog *orf2834* and *orf3045*, *orf3204* and *orf4555* (S5 Fig). Given the relevance of the global regulator H-NS in gene regulation, we sought to dissect further features of the Aar regulatory system on H-NS homologs in 042 by qRT-PCR (Fig 3). Levels of transcription of *orf1292* and *orf2834* were compared between 042*aar* and the wild type 042 strain. We observed 2–5 fold higher *hns* mRNA levels in 042 compared to the 042*aar* strain in the log phase (Fig 3A and 3B).

### Aar, the H-NS family and their four-way interaction circuit

**Fig 4:**
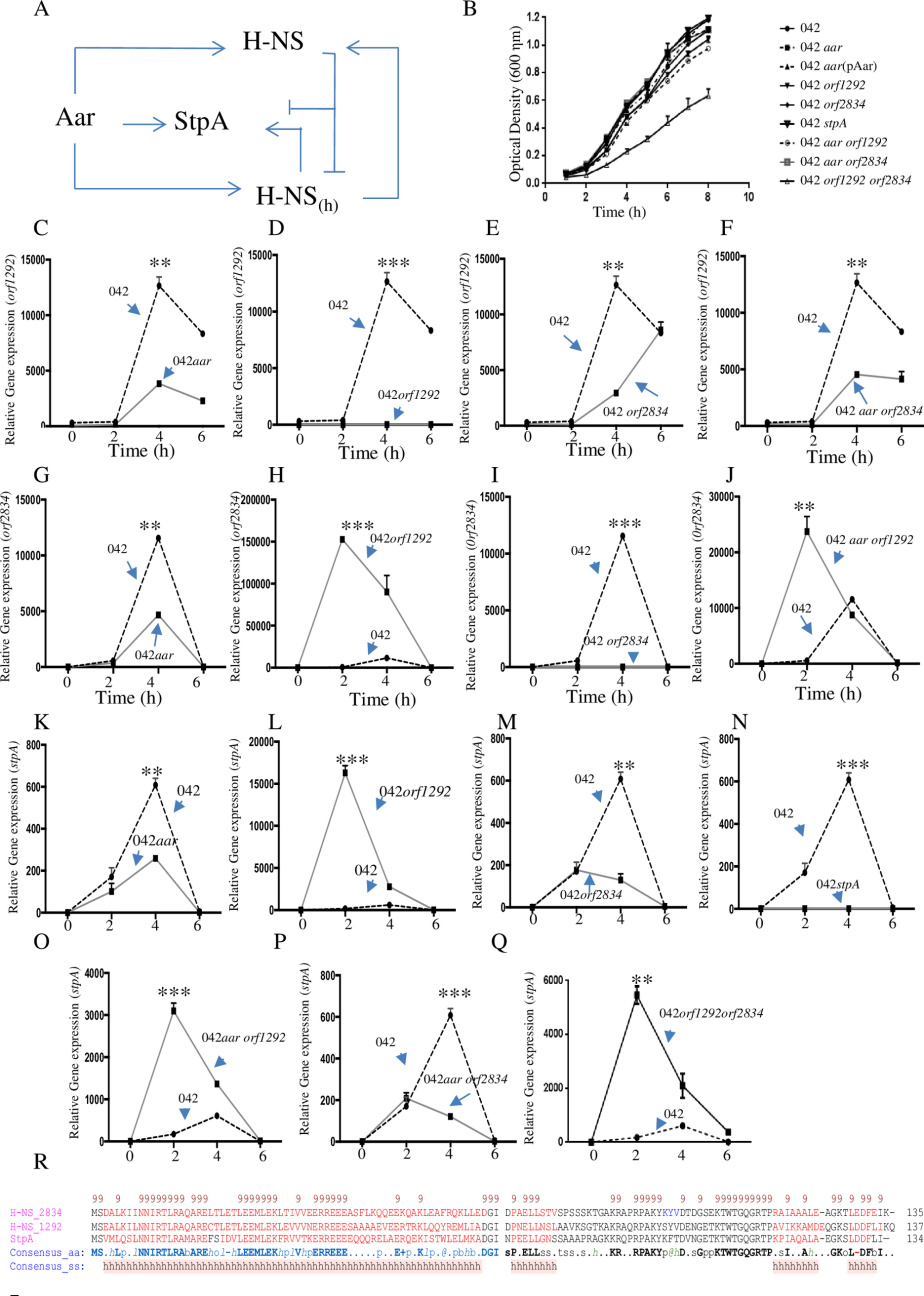
Aar, orf1292, orf2834 and StpA regulatory circuit. The H-NS homologs in 042 (Orf1292, Orf2834 and StpA) were compared by PROMALS3D algorithms (panel R). Single or double mutants in the HNS-like members were generated by λ-red technology. The bacterial growth was determined in 042 derivatives under Aar inducing conditions (panel B). Transcriptional profiles of *hns* genes were determined in the 042 HNS-like derivatives by qRT-PCR (panel C to Q). Illustrated model of H-NS—Aar circuit (panel A). Asterisks indicate significant difference by ANOVA (**, P < 0.001; ***, P < 0.0001).

The presence of multiple H-NS like members in the same bacterium has been reported [15,30,31]. In *E*. *coli* K12, StpA partially restores *hns* inactivation. In addition to H-NS and StpA, *Shigella flexneri* 2a encodes Sfh, a third member of H-NS like family [30]. EAEC 042 has three members of the H-NS-like family; H-NS (orf1292), H-NS_(h)_ (orf2834) and StpA. We applied a variety of prediction algorithms to reveal the presence of conserved secondary structure in the H-NS members. The PROMALS3D algorithm strongly predicted that the H-NS members have high similarity along the entire structure (70 to 80% similarity) (Fig 4R).

The structural similarities between members of the H-NS family suggests that Aar may be regulating not only H-NS and H-NS_(h)_ but also StpA in EAEC 042 in a four-way interaction circuit. To test this hypothesis, we generated a set of single and double *aar-hns* mutants in 042 (042*aar*, 042*orf1292*, 042*orf2834*, 042*stpA*, 042*aarorf1292*, 042*aarorf2834* and 042*orf1292orf2834*) (Fig 4). All the strains generated with the exception of 042*orf1292orf2834* showed no growth defect in Aar-inducing conditions (Fig 4B). We observed that not only *orf1292* and *orf2834* but also *stpA* were transcriptionally affected by Aar (Fig 4C, 4G and 4K). The most dramatic effect of Aar mutation was observed in the logarithmic phase of growth (4h), where 042*aar* strain showed reduced transcriptional levels for *orf1292* (3.4 fold), *orf2834* (2.47 fold) and *stpA* (2.35 fold) (Fig 4C, 4G and 4K). Transcriptomic analysis showed that deletion of either of the H-NS-like members alters the balance of the system. For example, deletion of *orf1292* drastically up-regulates the transcriptional expression of *orf2834* (268-fold compared to WT) (Fig 4H) and *stpA* (96-fold compared to WT) (Fig 4L) in the early phase of the logarithmic growth (2–6 h). Similar findings were observed in 042*aarorf1292* (*orf2834* and *stpA* were increased 42 fold and 18 fold respectively). The strong increase in the expression of StpA and H-NS_(h)_, may compensate for the detrimental effects of *hns* reduction in the absence of *aar*. We observed that deletion of *orf2834* reduced the transcriptional expression of *orf1292* and *stpA* as previously reported for its homolog *shf* in *Shigella flexnery* (Fig 4E and 4M) [30].

**Fig 5:**
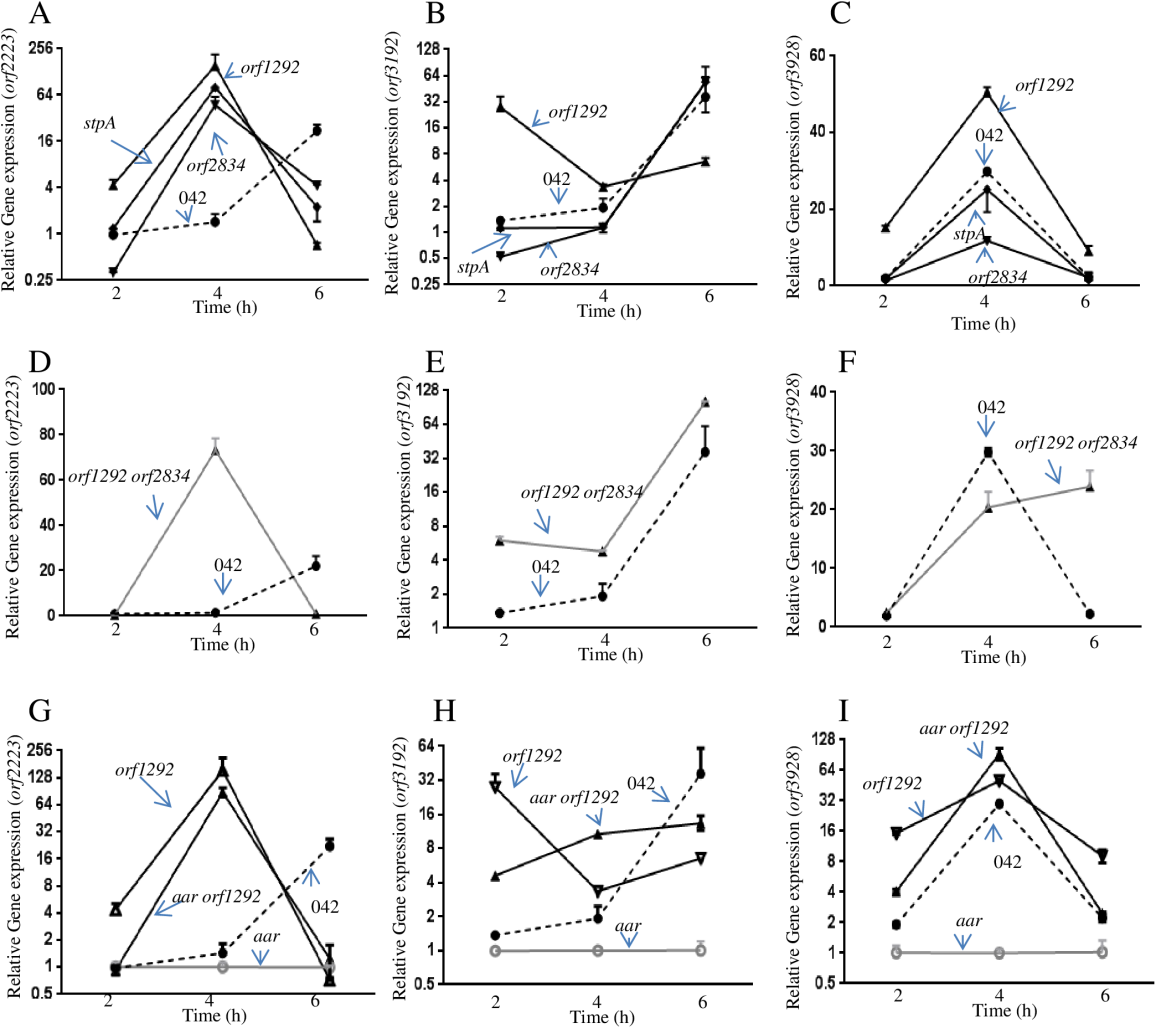
Transcriptional analysis of H-NS-Aar regulated genes. Transcriptional traits of Aar-regulated genes *orf2223* (panel A,D,G), *orf3192* (panel B,E,H) and *orf3928* (panel C,F,I) were evaluated in the H-NS derivatives by qRT-PCR.

Our finding supports the hypothesis that some of the effects of Aar on gene expression are likely through the modulation of the H-NS family in a four-way regulatory circuit (Aar, Orf1292 (H-NS), Orf2834 (H-NS_(h)_) and StpA) (Fig 4A). Therefore, all three H-NS homologs may have the ability to interact and modulate Aar-regulated genes. To test this hypothesize, we evaluate the transcriptional expression of three known Aar regulated genes (*orf2223*, *orf3192* and *orf3928*) in our collection of 042 derivatives (Fig 5). We observed that regulation of each gene was dependent on the bacterial growth phase, and on the concentration of Aar and H-NS-like members. Each analyzed gene showed different regulatory properties. However, we observed that Aar activity was dependent on H-NS members (Fig 5).

### Aar binds H-NS family

**Fig 6:**
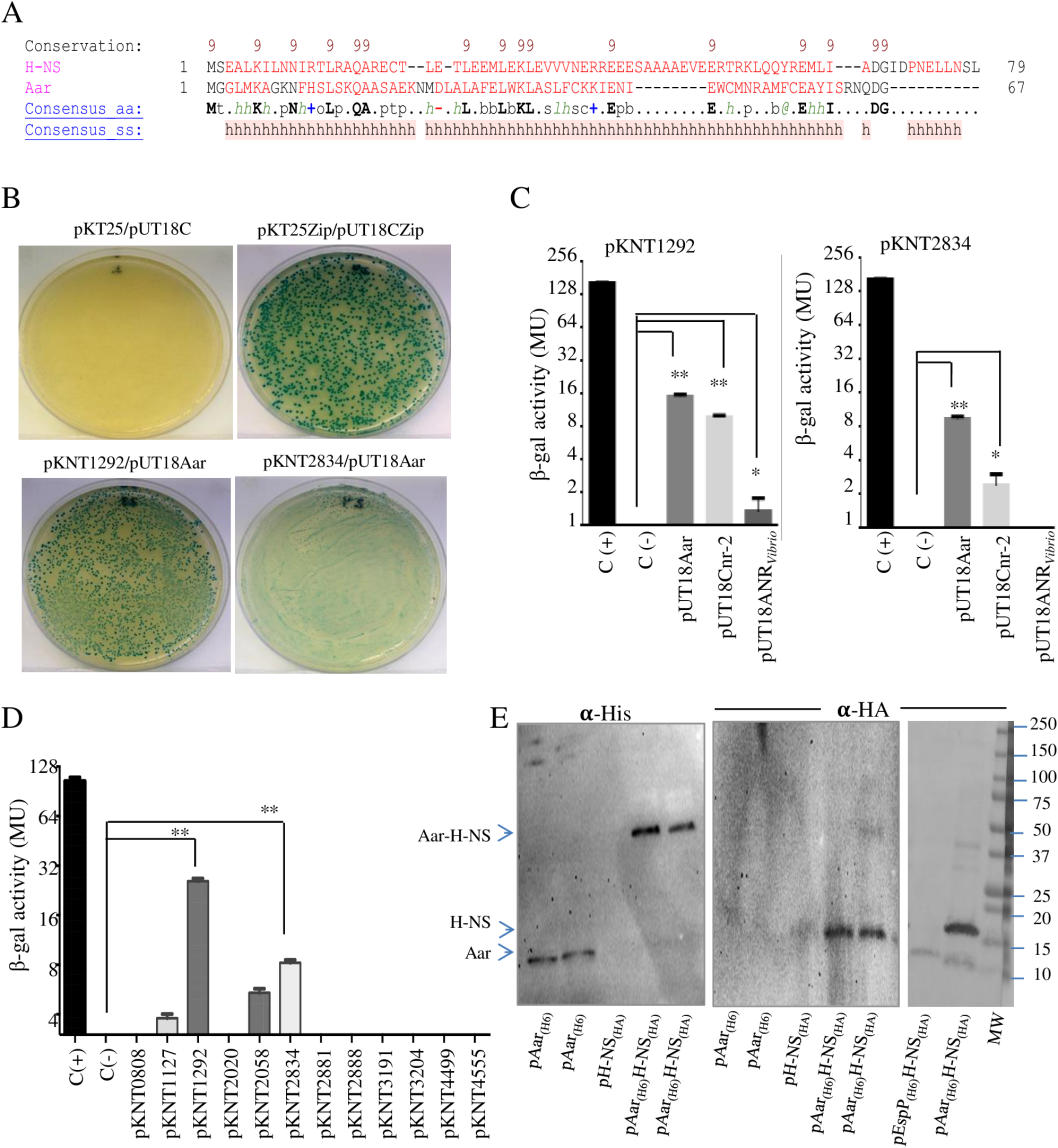
Aar binds Histone-like proteins. Secondary structure of N-terminal domain of orf1292 and Aar was compared by using Promals3d algorithms (panel A). Consensus predicted secondary structure symbols: alpha-helix: h; beta-strand: e. Aar—H-NS protein interaction was determined by using the BACTH bacterial two-hybrid system. pKNT derivatives encoding transcriptional factors were co-transformed with pUT18Aar into the reported *E*. *coli* BTH101 strain (Panel B, C and D). In panel C, H-NS (*orf1292*, *orf2834*)—ANR (Cnr-2 and ANR_*Vibrio*_) protein interactions were evaluated by two hybrid system technology. As controls, *E*. *coli* BTH101 strain was transformed either with empty vectors (pKNT25 and pUT18) (C(-), negative control) or the vectors encoding zip fragment (pKT25-zip and pUT18-zip) (C(+), positive control) respectively. β-galactosidase activity was determined in the assayed samples (panels B, C, and D). Aar-His, H-NS-HA and EspP-HA proteins were pull-down on cobalt resin, and analyzed by Western blotting assay using specific anti-HA and anti-H6 antibodies (Panel E). Data are representative of at least three independent experiments. Asterisks indicate significant difference by ANOVA (*, P < 0.01; **, P < 0.001).

Given that Aar does not have DNA binding domains, acts via direct binding to AggR and has structural similarity to the dimerization domain of H-NS (Fig 6A), we hypothesized that Aar might interact directly with H-NS family proteins. To test this hypothesis, we exploited the BACTH bacterial two-hybrid system, which has been used to detect protein-protein interaction of regulatory proteins in bacteria by us and other groups [2,32]. To perform this experiment, *aar* and *hns* genes (*orf1292* and *orf2834*) were fused to T25 and T18 fragments of the catalytic domain of *Bordetella pertussis* adenylate cyclase, expressed from plasmids pKNT25 and pUT18 respectively. The resulting plasmids were co-transformed into the reporter strain *E*. *coli* BTH101. We observed protein-protein interaction between Aar and H-NS manifested by the appearance of an intense to moderate green color on agar plates (Fig 6B). These qualitative observations were supported by quantification of β-galactosidase activity (Fig 6C). To verify the specificity of our system, twelve transcriptional factors (*orf0808*, *orf1127*, *orf1292*, *orf2020*, *orf2058*, *orf2834*, *orf2881*, *orf2888*, *orf3191*, *orf3204*, *orf4499*, and *orf4555*) were evaluated in the BACTH system (Fig 6D). The transcriptional factors were selected based on the transcriptome sequencing data (*orf1127*, *orf1292*, *orf2058*, *orf2834*, *orf3191*, *orf3204*, *orf4499*, and *orf4555*). Four unrelated transcriptional factors (*orf0808*, *orf2020*, *orf2881*, and *orf2888*) were also included in the study. Only 4 out of 12 transcriptional regulators revealed interaction, including *orf1292* and *orf2834*. As expected, the greatest levels of β-galactosidase activity were observed for *hns*-related genes *orf1292* and *orf2834*. Interestingly, we observed that *orf1127* (BssS, biofilm regulator) and *orf2058* (Transcriptional activator FlhD) were slightly able to interact with Aar in this assay (Fig 6D).

The molecular interaction between H-NS and Aar was also modeled and assembled by TM-Score (TM-score 0.2793 and 0.3555) [33], RasMol [34] and the UCSF Chimera package [35]. The second α-helix of Aar was predicted to interact with the oligomerization domain of H-NS (S10 Fig).

If interaction with H-NS is an important regulatory feature of the ANR family, then ANR-H-NS protein-protein interaction should also be demonstrated with other members of the ANR family. ANR_*Vibrio*_ and Cnr-2 (of ETEC) have been shown to rescue Aar activity in the 042*aar* strain [2]. Using the BACTH system, we observed protein-protein interaction between Cnr-2 and both *orf1292* and *orf2834* H-NS regulators (Fig 6C). *ANR*_*Vibrio*_ showed binding only to *orf1292* but not to *orf2834* (Fig 6C).

To confirm interaction of Aar and H-NS proteins *in vivo*, we performed a pull-down assay (Fig 6E). *E*. *coli* K12 transformed with pAar_(H6), p_H-NS_(HA)_ or _p_Aar_(H6)_H-NS_(HA)_ plasmids, expressing His-tagged Aar, HA-tagged H-NS or both, respectively, were cultivated overnight at 37°C. As a control of the pull-down assay, *E*. *coli* K12 was also transformed with pEspP_(H6)_ [36] and _p_H-NS_(HA)_ (S11 Fig)_._ The samples were sonicated and the supernatants incubated with cobalt resin. The samples were separated in SDS-PAGE gels and analyzed by western blot with specific antibodies against the protein-tags H6 and HA. Our data show that H-NS_(HA)_ is pulled-down only in presence of Aar_(H6)_ but not with the EspP_(H6)_ protein (Fig 6E).

### Aar modulates H-NS DNA binding properties

**Fig 7:**
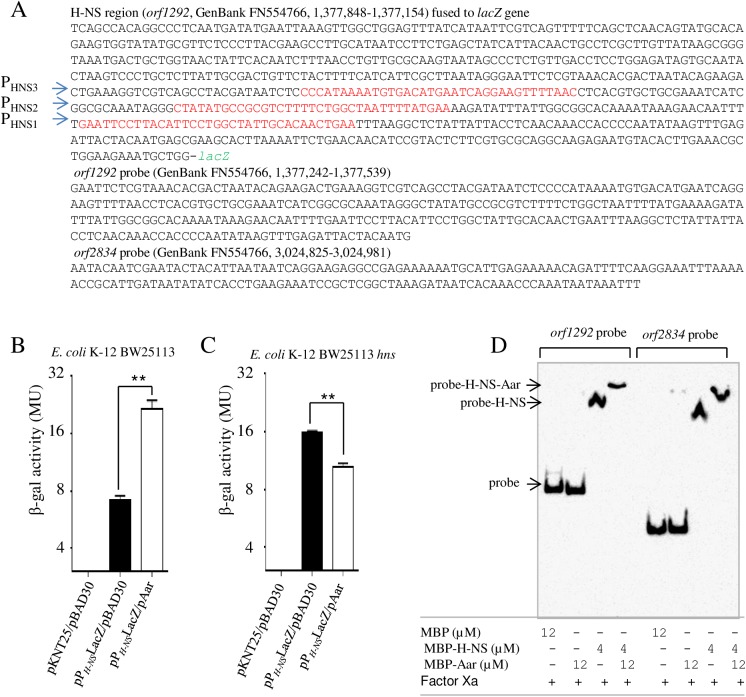
Aar modulates transcriptional levels of H-NS. Regulatory region of H-NS (*orf1292*, region 1,377,848–1,377,154, GenBank FN554766) was cloned into pEF-ENTR-lacZ plasmid (panel A). H-NS bindings sites are indicated in red. The resulting plasmid pP_*H-N*_SLacZ was co-transformed with the pAar plasmid in *E*. *coli* K-12 BW25113 (panel B) and BW25113*hns* mutant strains (panel C). β-Galactosidase assays were performed accordingly to the method of Miller. *orf1292* and *orf2834* probes were evaluated by EMSA using 5’ biotinylated probes (Panel A). Probes were incubated with either MBP-Aar, MBP-H-NS or both, in the presence of factor Xa (Panel D) and analyzed as indicated in material and methods. Data are representative of at least three independent β-Galactosidase assays. Asterisks indicate significant difference by ANOVA (**, P < 0.001).

Protein-protein interaction of Aar-H-NS and dependence of *hns* expression on *aar* was unexpected, and provides a potential mechanism for global effects of Aar on gene expression in EAEC. We sought to confirm this relationship using a LacZ reporter system. The regulatory region of H-NS (*orf1292*, region 1,377,848–1,377,154) was cloned into plasmid pEF-ENTR-lacZ (Fig 7A). *E*. *coli* K-12 BW25113 and an *E*. *coli* K-12 BW25113 *hns* mutant were co-transformed with pP_H-NS_LacZ and pAar plasmids. Higher levels of β-galactosidase activity were detected in the parental *E*. *coli* K-12 BW25113 strain in the presence of Aar (~3 fold) and were dependent on the presence of H-NS (Fig 7B and 7C).

We hypothesized that Aar-H-NS interaction would modify H-NS binding to H-NS regulated promoters, consequently altering H-NS-mediated negative regulation. To test this hypothesis, we performed EMSA experiments using 5’ biotinylated H-NS *orf1292* and *orf2834* probes (GenBank, FN554766, 1,377,242–1,377,539 and 3,024,825–3,024,981 regions) (Fig 7A). Probes were incubated with either MBP-Aar, MBP-H-NS or both, in the presence of factor Xa as described in Materials and Methods. The purified native form of Aar is insoluble and requires the use of MBP for its expression [37]. We observed that Aar and H-NS interacted only when MBP was removed with factor Xa. Only H-NS and H-NS/Aar, but not Aar bound to these probes (Fig 7D). Intriguingly, we found that when both H-NS and Aar were incubated together the probes super-shifted, suggesting binding of Aar to the H-NS-probe complex (Fig 7D).

**Fig 8:**
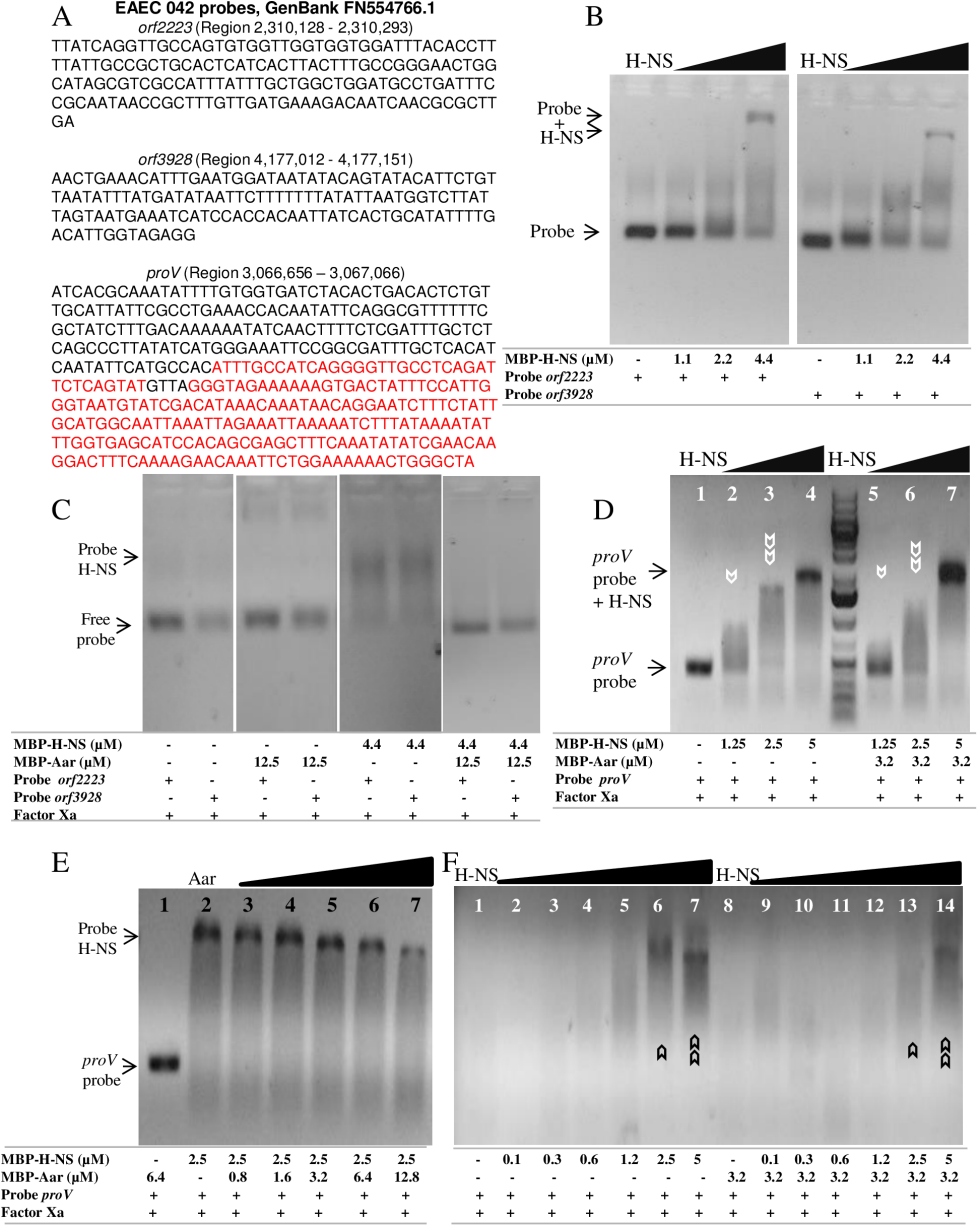
Aar modulates DNA-binding properties of H-NS. *orf2223* and *orf3928* probes (Panel A) were incubated with different concentration of H-NS protein and analyzed by EMSA (Panel B). H-NS binding sites are indicated in red. In a second experiment, the samples were incubated with either H-NS, Aar or both proteins and analyzed by EMSA (Panel C). In parallel, *proV* was incubated with H-NS in either presence or absence of Aar. The presence of Aar abolished the H-NS-DNA interaction (panel D, white arrows, lanes 2, 3, 5, 6). A similar approach was used to evaluate the effect of using increasing concentrations of Aar (0.8–12.8 μM) (panel E, lanes 3 to 7). DNA probe and H-NS samples were incubated in either presence or absence of Aar and treated with DNase I. Incubation of samples with Aar (3.2 μM) disrupted DNA protection and increased DNase I effect on *proV* probe (panel F, black arrows, lanes 6, 7, 13, 14).

Given that the regulation of gene expression by H-NS is modulated by variations in the number and organization of binding sites [18,38], we hypothesized that Aar may affect binding of H-NS to DNA more avidly in some promoters than others. To test this hypothesis, we performed EMSA experiments using promoters of three different HNS-regulated genes, two of which were previously evaluated in this study (*orf2223* and *orf3928*) (Fig 5) and *proV*. The latter is a well-characterized H-NS-regulated gene (H-NS binding regions of the *proV* DNA are depicted in red in Fig 8A [39]). We demonstrated that H-NS binds to *orf2223* and *orf3928* probes by EMSA as shown in Fig 8B. As expected, Aar does not bind to these probes but alters H-NS DNA binding at different levels as judged by the presence of free probes and probe-shift patterns in EMSAs (Fig 8C and 8D). Aar inhibited H-NS binding to *orf2223* and *orf3928* DNA probes (Fig 8C), but only modified the H-NS binding pattern to *proV* probe, shown at various H-NS concentrations (1.25–5 μM) (Fig 8D). To further disrupt the H-NS-*proV* DNA interaction with Aar, we performed EMSA using increasing concentration of Aar (0.8–12.8 μM) and a constant concentration of H-NS (2.5 μM). In these conditions, we observed that the H-NS-*proV* probe complex bands become progressively diminished with increasing concentrations of the Aar protein, however we did not observe free-probe species (Fig 8E). We also performed an Aar DNA protection assay. Target DNA was incubated for 30 min with increasing concentrations of H-NS in the presence or absence of Aar. The samples were treated with DNaseI for 30 min and the enzyme was inactivated at 75°C for 10 min, followed by EMSA analysis as indicated in materials and methods. We showed that incubation of the DNA-HNS complex with Aar increased susceptibility of DNA to degradation by DNaseI (Fig 8F). Together, these findings suggest a differential effect of Aar on H-NS binding to HNS-regulated promoters.

### Aar affects H-NS expression *in vivo*

**Fig 9:**
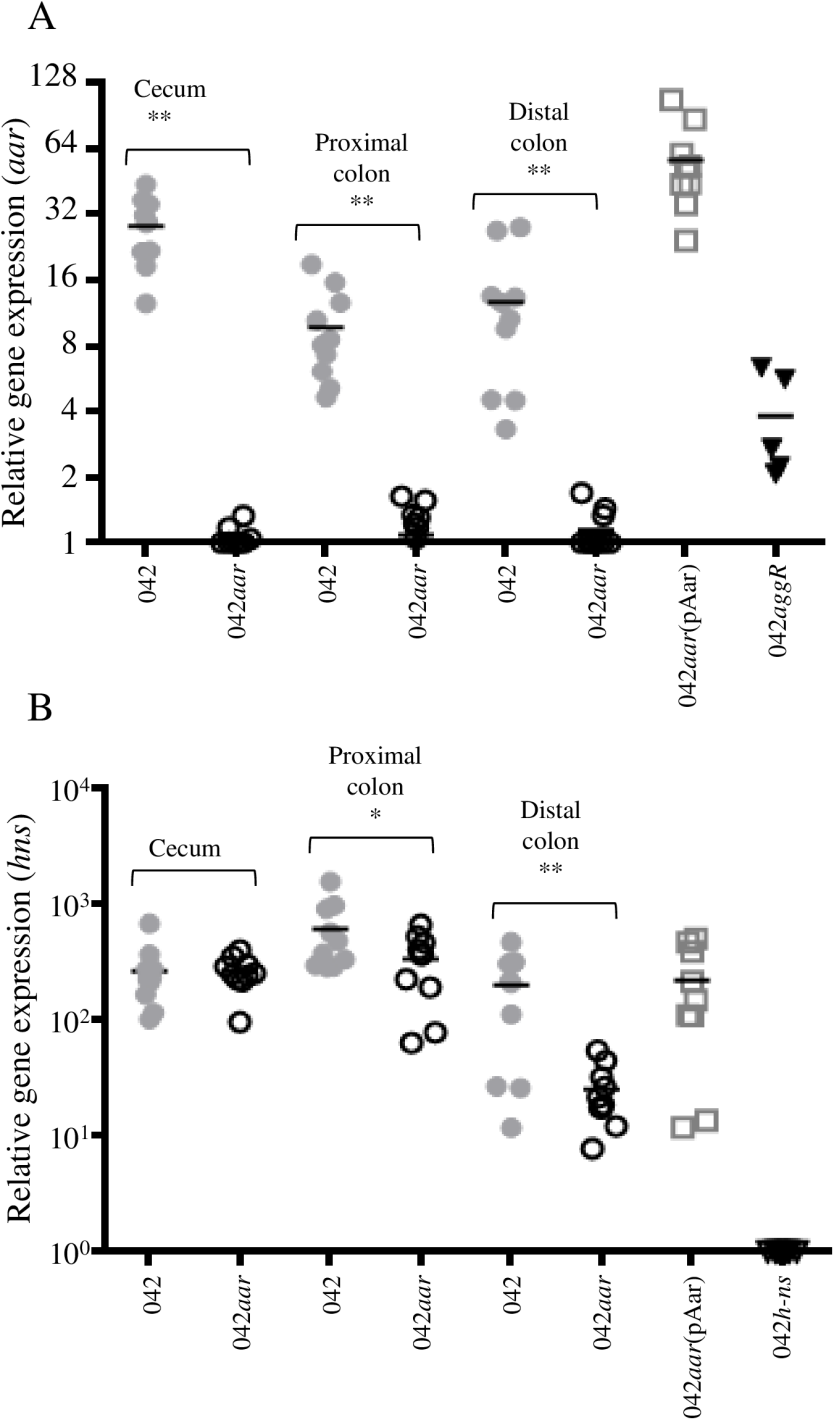
Aar affects H-NS expression *in vivo*. The streptomycin-treated mouse model was used evaluate the Aar-H-NS regulatory system in vivo. Groups of 5–10 mice were inoculated with 0.2X10^10^ CFU of 042 derivatives. *aar* (Panel A) and *hns* (Panel B) transcription was ascertained by qRT-PCR in fecal samples from cecum, proximal and distal colon. As negative controls, mice were inoculated with either 042*aggR* or 042*hns*. Complementation *in trans* of 042*aar* was evaluated in the proximal colon samples. Data are representative of two independent experiments. Asterisks indicate significant difference by ANOVA (*, P < 0.01; **, P < 0.001).

We sought to dissect further features of the Aar-H-NS regulatory system in the streptomycin-treated mouse model, which has been used previously to study gene regulation *in vivo* for this pathogen [40]. Groups of 10 mice were inoculated with 0.2 X 10^10^ CFU of 042, 042*aar*, and 042*aar*(pAar). As a control, groups of 5 mice were inoculated with either 042*aggR* or 042*hns*. No differences in bacterial shedding were observed between the groups at 24 h post-inoculation. Mice were euthanized 24 h post-bacterial inoculation. *aar* and *hns* transcription was ascertained by qRT-PCR in fecal samples from different intestinal compartments. Notably, *aar* was highly expressed in the intestinal lumen (3–50 fold) in the early stages of infection. *aar* expression was higher in cecum than in colon (Fig 9A). We observed that the expression of *aar* was dependent on AggR; *aar* expression in the 042*aggR* mutant was reduced *in vivo* (2–4 fold) (Fig 9A, triangles). H-NS expression was substantially reduced in the colon in 042*aar* (~10 fold) compared to the parent strain but not in cecum (Fig 9B). Taken together, we provide evidence that Aar is expressed *in vivo* and is required for maximal expression of H-NS.

## Discussion

The bacterial chromosome is compacted to fit in the bacterium by histone-like proteins [41,42]. Spatial and temporal expression of virulence factors depends on the controlled removal of global histone-like regulators from the chromosome. H-NS, a member of this family, forms high molecular weight complexes and silences gene expression through promoter occlusion and shaping of DNA [23–26]. H-NS members are well recognized as negative regulators of 5 to 10% of the bacterial genome [17,18]. *hns* mutation in uropathogenic *E*. *coli* resulted in increased expression of H-NS-regulated fimbriae (SfaA and PrfA), iron uptake systems, and genes involved in stress adaptation [43]. H-NS has been shown to silence expression of genes with low GC content; we have reported that genes under AggR control have this feature [31,44].

In this study, we demonstrated that *aar* mutation in EAEC affects the expression of more than 200 genes. Some of the effects of Aar on gene expression are likely through the expression of histone-like family (Fig 4), including LpfC fimbriae, LPS-related enzymes *orf3928*, *orf3931* and *orf3932*, genes involved in stress adaptation (*gadXW* transcriptional factors), and porins (Fig 2). This observation in conjunction with two-hybrid system and pull-down assays (Fig 6), *lacZ* fusion assays (Fig 7), and EMSA studies (Figs 7 and 8), confirmed that Aar exerts global regulatory activity at least in part via H-NS, and that this effect may be due to direct binding of the three proteins present in EAEC 042 strain.

We have shown that Aar binds to H-NS and alters its binding to H-NS regulated promoters in different ways, perhaps depending on the presence of low or high affinity H-NS binding sites. Similar H-NS-DNA shift patterns have been seen with other small regulatory proteins such as the family of naturally occurring truncated H-NS derivatives lacking the DNA-binding domain, termed H-NST family in pathogenic *E*. *coli* strains [45], and the *5*.*5* protein of bacteriophage T7 [46]. Detailed analysis of these proteins shows that, although they do not avoid the binding of H-NS to DNA upon interaction with H-NS, they affect the high order oligomerization of H-NS and exhibit a potent anti-H-NS function with global effects [45,46].

**Fig 10:**
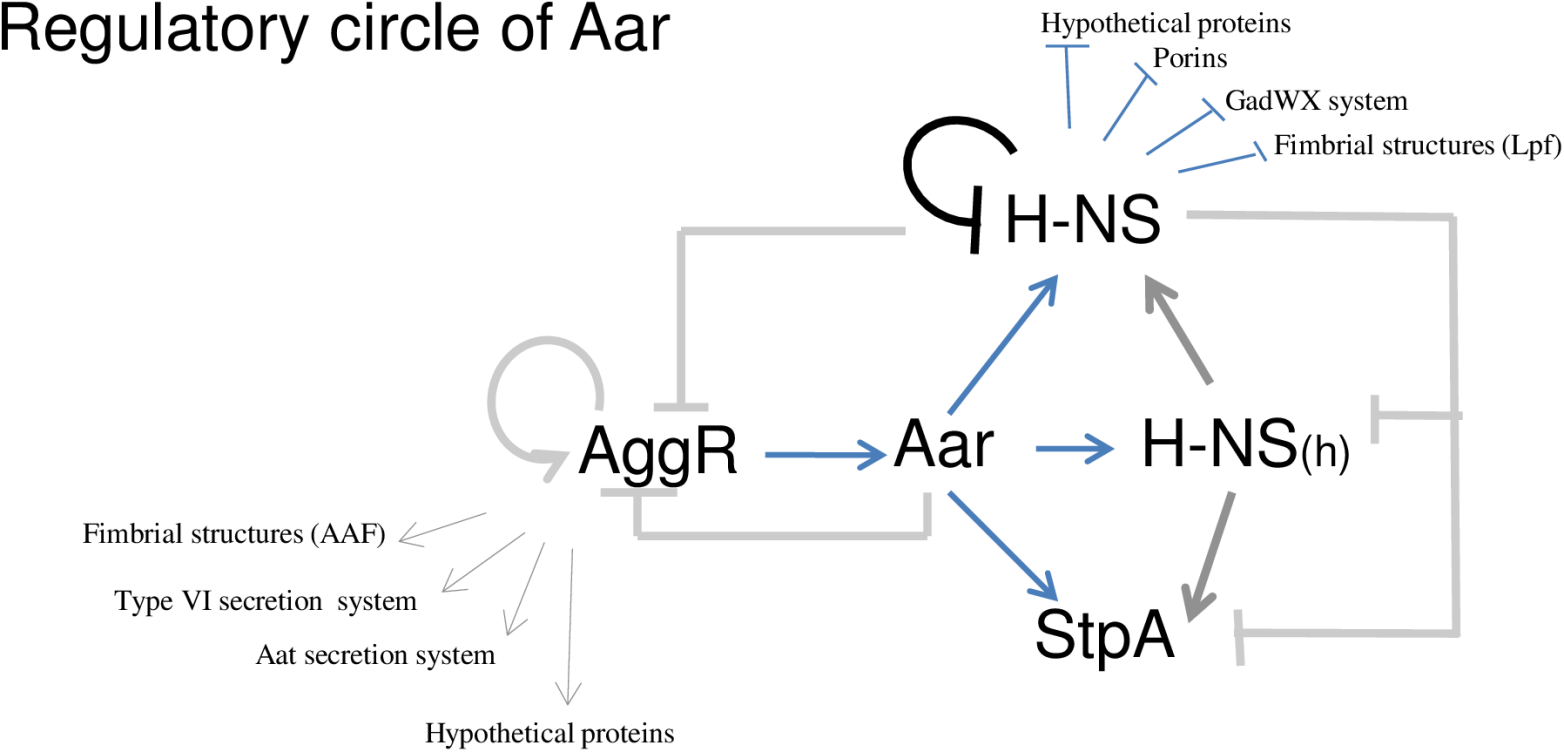
Model of Aar regulation in EAEC strain 042.

It is tempting to hypothesize that the outcomes of Aar/H-NS and Aar/AraC-like regulator interactions assure a concerted expression of fitness and virulence factors that prepare the bacteria for colonization (Fig 10). During the initial stage of the infection, H-NS (an abundant protein at 20,000 copies per cell) is intimately associated with DNA, including genes in the AggR regulon. Upon AggR activation, Aar could act to lift H-NS silencing of the regulon, thereby augmenting the effects of AggR. Paradoxically, H-NS acts to repress its own expression, and therefore binding of Aar to H-NS could increase expression of the latter protein, possibly thereby diminishing expression of the AggR regulon. Our data revealed that Aar affects up to 4.0% of bacterial genes under the conditions tested (Fig 1), and therefore paradoxical, nuanced control of such a large regulon could be beneficial to the bacterium.

Also remarkable is the fact that Aar modifies the activity of three additional H-NS members. It is tempting to hypothesize that the net effects of Aar may be enhanced by affecting three partners of the same family. Additional studies are underway to dissect the contributions of these related H-NS proteins. H-NS is required for virulence in pathogenic bacteria including uropathogenic *E*. *coli*, *Salmonella typhimurium*, and *Vibrio cholerae* [19,43,47,48]. Here we report that Aar regulates H-NS activity in EAEC, and that H-NS is highly expressed in the intestinal lumen in early stages of the infection. Expression of H-NS is significantly decreased in an 042*aar* mutant in the colon, suggesting a role for both ANR and H-NS families in the context of infection. Taken together, our findings suggest that ANR is a common, highly conserved mechanism of regulation of bacterial virulence *in vivo*.

## Materials and methods

### Bacterial strains and growth conditions

Bacterial strains, plasmids and primers used in this study are shown in S1 and S2 Tables. The nomenclature used by NCBI (http://www.ncbi.nlm.nih.gov) to describe genomic sequences in EAEC042 (FN554766) or genes in pAA plasmid (FN554767) was applied in this study. Putative gene assignments and homologies are listed in Table 1. Strains EAEC 042, 042pAA-, 042*aggR*, and 042*aar* were previously described [1,10,49,50]. In this study, 042 derivatives were generated by lambda red technology [51]. The locus for *orf1292* (1,376,681–1,377,424), *orf2834* (3,024,998–3,025,605), and *stpA* (3,058,170–3,058,930) in 042 (GenBank FN554766.1) were replaced with the kanamycin (km) resistance marker. 042 derivatives were identified by PCR using specific primers for *orf1292*, *orf2834* and *stpA* (S2 Table). Bacterial cultures were routinely propagated in Luria Broth (LB) and Dulbecco’s modified Eagle’s medium with 0.4% glucose (DMEM high glucose) (Gibco) as previously described [13].

### RNA-seq

To examine the transcriptome in an unbiased manner, RNA-seq analysis was performed. RNA was extracted from EAEC 042 derivatives grown in DMEM-Glucose. RNA was extracted with TRIzol (Invitrogen) and treated with RNase-free DNase set (Qiagen) to remove contaminating DNA. The samples were purified in RNeasy Mini kit columns (Qiagen) and used for library construction. The RNA samples were converted into cDNA libraries using the Ovation Prokaryotic RNA-Seq System (NuGen) and sequenced on the Illumina HiSeq 2000 to generate 100 bp paired-end reads at the Institute for Genome Sciences (http://www.igs.umaryland.edu/resources/grc/analysis.php). Reads were mapped to the EAEC 042 chromosome and pAA plasmid with the BWA aligner [52]. Counts for each annotated genomic feature were determined by htseq-count (http://htseq.readthedocs.io/en/release_0.9.0/). Differential expression between counts for each feature was then calculated with DESeq [53] using the false-detection rate-adjusted Benjamini Hochberg P value. The fold change of differentially expressed genes vs. P value was plotted by using the GraphPad Prism 6 (GraphPad Software, Inc., CA, USA).

### Cloning and purification of recombinant proteins

H-NS was cloned into pMAL-c5x plasmid (New England Biolabs) and expressed as fusion proteins with the maltose binding protein (MBP). Aar-MBP protein was purified as published before [2]. H-NS-MBP was expressed in *E*. *coli* NEB Express (New England Biolabs) at 37°C. Cells were grown in 1 liter of LB to an OD_600_ of 0.6 and induced for 3 h with 0.3 mM IPTG. The bacteria were harvested by centrifugation, and bacterial pellets were resuspended in 25 ml column buffer (20 mM Tris-HCL, 200 mM NaCl, 1 mM DTT, and 1 mM EDTA, pH 7.5), and lysed by sonication on ice. Bacterial preparations were centrifuged and cleared lysates were loaded onto an amylose resin column (New England Biolabs), washed with 5 volumes of column buffer, and eluted with column buffer containing 10 mM maltose. Pure protein preparations of H-NS-MBP were dialyzed overnight in PBS.

### Electrophoretic mobility shift assay (EMSA)

Direct binding of H-NS and effects of Aar in the DNA binding activity to *orf2223*, *orf3928* and *proV* regulatory region were evaluated by EMSA as previously described [39]. Aar and H-NS were expressed as fusion proteins with the maltose binding protein (MBP). The proteins were purified and the MBP tag was cleaved with 1% of Factor Xa as previously reported [2]. Probes (1μg) were amplified by PCR (Genbank, FN554766, region 3,066,656–3,067,066), purified and incubated with H-NS. The samples were prepared in 20–50 μl reaction mixtures containing 10mM Tris-HCl (pH 7.5), 1 mM Na-EDTA, 80 mM NaCl, 10 mM β-mercaptoethanol, and 4% glycerol. Samples were incubated for 30 minutes a 37°C in either the presence or absence of Aar.

In parallel, 5’biotinylated probes for *orf1292* and *orf2834* were amplified by PCR. EMSAs were performed as previously described [2], using the reaction mixtures described above. Following electrophoresis, the gel was incubated in a denaturing solution (1.5 M NaCl, 0.5 M NaOH) for 30 min, washed in water, and washed twice in a neutralizing solution (1.5 M NaCl, 0.5 M Tris-HCl pH 7.2, 1 mM EDTA) for 15 min. Samples were transferred to a Zeta-probe membrane, and probes were visualized using the Chemiluminescent Nucleic Acid Detection kit (Thermo Scientific).

For the DNase I protection assays, after protein-DNA complex formation, the samples were incubated with 25 ηg of DNase I (RNase-free DNase set) (Qiagen) for 30 min. The DNase I was inactivated at 75°C for 10 min. The samples were resolved on 1.2% agarose gels with 0.5 x TBE (Tris-borate-EDTA buffer) as the running buffer and stained with ethidium bromide.

### Pull-down experiments

*E*. *coli* K-12 transformed with plasmid expressing Aar and H-NS proteins (pAar_(H6)_H-NS_(HA)_, pAar_(H6)_, pH-NS_(HA)_ and pEspP_(H6)_H-NS_(HA)_) were grown in LB to OD_600_ nm of 0.4. Expression of Aar and H-NS was induced with 2% of arabinose overnight at 30°C. The bacteria culture was treated with 5% formaldehyde and incubated for 10 min before quenching with PBS-glycine (0.125 M final glycine concentration). The bacterial cultures were pelleted, washed, and resuspended in 6 ml of lysis buffer (50 mM of sodium phosphate, 300 mM sodium chloride, 10 mM imidazole, 10 μM β-mercaptoethanol, 5% glycerol). The bacterial suspension was sonicated for 2 min at 22 μm amplitude. The procedure was repeated until the solution change color to translucent. Bacterial preparations were centrifuged and 600 μl of cleared lysates were incubated overnight with cobalt resin. The proteins were purified following the manufacture specifications (Thermo Fisher Scientific), analyzed by SDS-PAGE and confirmed by Western blotting assay using specific anti-HA and anti-H6 antibodies (Thermo Fisher Scientific).

### Bacterial adenylate cyclase two-hybrid system (BACTH)

The genes for *orf0808*, *orf1127*, *orf1292*, *orf2020*, *orf2058*, *orf2834*, *orf2881*, *orf2888*, *orf3191*, *orf3204*, *orf4499* and *orf4555* were amplified by PCR, digested with *Bam*HI/*Eco*RI and cloned into pKNT25 plasmid. The plasmids were analyzed by PCR and sequenced at the University of Virginia DNA Science Core. Plasmids pKT25/pUT18C and pKT25Zip/pUT18CZip were used as experimental negative and positive controls, respectively. The plasmids and primers used in this work are listed in S1 and S2 Tables. The plasmids were purified and cotransformed into the reporter strain *E*. *coli* BTH101. Colonies were selected on LB agar plates containing carbenicillin (100 μg/ml), kanamycin (50 μg/ml), 5-bromo-4-chloro-3indolyl-β-d-galactopyranoside (X-Gal) (40 μg/ml), and isopropyl-β-d-thiogalactopyranoside (IPTG) (1 mM).

### β-Galactosidase assays

*E*. *coli* BTH101 was cotransformed with pUT18 and pKNT25 derivatives encoding ANR and regulatory proteins. The clones were grown at room temperature for 48–72 h in LB plates with 1 mM IPTG. β-Galactosidase assays were performed accordingly to the method of Miller. Briefly, bacterial samples were suspended in 1 ml of Z buffer (60 mM Na_2_HPO_4_·7H2O, 40 mM NaH_2_PO_4_·H2O, 10 mM KCl, 1 mM MgS0_4_·7H_2_O, 50 mM β-mercaptoethanol), 20 μl of 0.1% SDS and 40 μl of chloroform. 100 μl of sample was incubated with 20 μl of ONPG (4 mg/ml) for 2 min at room temperature. The reaction was terminated by the addition of 50 μl of 1 M Na_2_CO_3_. Samples were diluted in 800 μl of Z buffer. Optical densities at 420 ηM, 550 ηM and 600 ηM were determined. β-galactosidase activity was calculated by using the Miller formula (Miller unit = 1000 x (Abs_420_ - (1.75 x Abs_550_) / T x V x Abs_600_); T, reaction time; V, volume of culture assayed in milliliter).

For the H-NS regulatory region fused to the LacZ reporter system, H-NS region (GenBank, FN554766, *orf129*2 region; 1,377,848–1,377,154) was amplified by PCR digested with *Nhe*I and *Bam*HI and cloned into pEF-ENTR-lacZ plasmid to generate pP_*H-NS*_LacZ. The pEF-ENTR-lacZ plasmid was a gift from Eric Campeau (Addgene Plasmid #17430) [54]. *E coli* K-12 BW25113 (keio parental strain) and *E*. *coli* K-12 BW25113 *hns* (Keio *hns* knockout) [55] were cotransformed with pP_*H-NS*_LacZ and pAar. As controls, the strains were transformed with empty pKNT15 and pBAD30 plasmids. The strains were grown at 37°C, 1 ml of bacteria was pelleted and suspended in 1 ml of Z buffer and prepared for LacZ assay as indicated above.

### qRT-PCR

qRT-PCR analysis was performed to corroborate the microarray data. Briefly, overnight bacterial cultures of EAEC were diluted 1:100 into 13 ml of DMEM high glucose (*aar*-inducing conditions), and incubated at 37°C with shaking for 5 h. Samples were collected at various time points along the log phase of growth. Extraction of RNA, cDNA synthesis and qRT-PCR assays were performed as previously described [13]. Reactions were run in experimental duplicate using two independent cDNA preparations. Expression levels for each queried gene were normalized to the constitutively expressed *cat* gene in EAEC 042.

### Streptomycin-treated mouse model

Groups of 5–10 male BALB/c mice, 5 wks old (Jackson Laboratories) were provided with drinking water ad libitum containing 5 g/liter streptomycin for 24 h prior to bacterial inoculation. The inoculation strains (042, 042*aar*, 042*aar*(pAar), 042*hns* and 042*aggR*) were grown overnight in LB broth, diluted 1:100 and incubated in DMEM-high glucose for 5 h. Bacteria were pelleted and adjusted to 0.2 X 10^10^ cfu/ml. Mice were orogastrically inoculated with 0.2 ml of the inoculum and euthanized 24 h post-inoculation. Cecum, proximal and distal colon compartments were excised. The intestinal compartments were kept in 1 ml of RNA later stabilization solution (Thermo Fisher Scientific). The samples were homogenized and filtered on sterile gauze pads. The samples were pelleted and resuspended in 1ml of TRIzol (Invitrogen). Extraction of RNA, cDNA synthesis and qRT-PCR assays were performed as previously described [13]. Reactions were run in experimental duplicate. Expression levels for each queried gene were normalized to the constitutively expressed *cat* gene in EAEC 042.

### Ethics statement

Animal experiments were performed in accordance with the Guide for the Care and Use of Laboratory Animals of the National Institutes of Health and with the permission of the American Association for the Assessment and Accreditation of Laboratory Animal Care. The protocol was reviewed and approved by the Institutional Animal Care and Use Committee of the University of Virginia (Protocol No. 3999).

### Bioinformatic and statistical analysis

Protein secondary structures were analyzed by using Promals3d algorithms http://prodata.swmed.edu/promals3d/promals3d.php. The sequences for ANR homologs and H-NS homologs were obtained from NCBI. Statistical analysis of the data for β-galactosidase assays, qRT-PCR, and mice experiments was performed by using the GraphPad Prism 6 (GraphPad Software, Inc., CA, USA). The statistical significance of the differences in the sample means was calculated by using ANOVA with post hoc Tukey’s correction. Results were considered significant at P < 0.05. The CG profile of pAA plasmid was generated by using GC-Profile algorithms (http://tubic.tju.edu.cn/GC-Profile/). H-NS–Aar interaction was also modeled and assembled by TM-Score [33], RasMol [34] and the UCSF Chimera package [35].

## Supporting information

S1 Fig Hypothetical proteins. Differentially expressed genes detected by using RNA-seq analysis (p<0.05). Genes for EAEC strain 042 vs 042*aar* (panel A) or 042*aar* vs 042*aar*(pAar) (panel B). AggR-regulated genes are indicated in yellow.
(PPTX)

S2 Fig Transporter proteins. Differentially expressed genes detected by using RNA-seq analysis (p<0.05). EAEC strain 042 vs 042*aar* (panel A) or 042*aar* vs 042*aar*(pAar) (panel B) are showed in the graphs. AggR-regulated genes are indicated in yellow.
(PPTX)

S3 Fig Metabolic functions. Differentially expressed genes detected by using RNA-seq analysis (p<0.05). EAEC strain 042 vs 042*aar* (panel A) or 042*aar* vs 042*aar*(pAar) (panel B) are showed in the graphs.
(PPTX)

S4 Fig Related virulence factors. Differentially expressed genes detected by using RNA-seq analysis (p<0.05). EAEC strain 042 vs 042*aar* (panel A) or 042*aar* vs 042*aar*(pAar) (panel B) are showed in the graphs. AggR-regulated genes are indicated in yellow.
(PPTX)

S5 Fig Global regulators and transcriptional factors. Differentially expressed genes detected using RNA-seq analysis (p<0.05). EAEC strain 042 vs 042*aar* (panel A) or 042*aar* vs 042*aar*(pAar) (panel B) are showed in the graphs. AggR is indicated in yellow.
(PPTX)

S6 Fig Outer membrane proteins. Differentially expressed genes detected by using RNA-seq analysis (p<0.05). EAEC strain 042 vs 042*aar* (panel A) or 042*aar* vs 042*aar*(pAar) (panel B) are showed in the graphs.
(PPTX)

S7 Fig Phage proteins. Differentially expressed genes detected by using RNA-seq analysis (p<0.05). EAEC strain 042 vs 042*aar* (panel A) or 042*aar* vs 042*aar*(pAar) (panel B) are showed in the graphs.
(PPTX)

S8 Fig Other functions. Differentially expressed genes detected by using RNA-seq analysis (p<0.05). EAEC strain 042 vs 042*aar* (panel A) or 042*aar* vs 042*aar*(pAar) (panel B) are showed in the graphs. AggR-regulated gene is indicated in yellow.
(PPTX)

S9 Fig Analysis of CG content of Aar-regulated region. (PPTX)

S10 Fig Hypothetical model of H-NS and Aar interaction. The molecular interaction between Aar (Panel A) and H-NS (Panel C) of *E*. *coli* (1N18) (panel B), or Aar and H-NS (Panel E) of *S*. *typhimurium* (1LR1) (panel D) was modeled by TM-score, RasMol and the UCSF Chimera package. The second α-helix from Aar was predicted to overlap with the H-NS oligomerization domain.
(PPTX)

S11 Fig Pull-down samples. *E*. *coli* K-12 lysates were analyzed by SDS-PAGE and used in the pull-down assay.
(PPTX)

S1 Table Strains and plasmids used in this study. (DOCX)

S2 Table Primers used in this study. (DOCX)

S3 Table RNA-seq data for 042 vs 042*aar*, chromosomal genes. (DOCX)

S4 Table RNA-seq data for 042*aar* vs 042*aar*(pAar), chromosomal genes. (DOCX)

S5 Table RNA-seq data for 042 vs 042*aar*, pAA genes. (DOCX)

S6 Table RNA-seq data for 042*aar*(pAar) vs 042*aar*, pAA genes. (DOCX)
