# Supplementary material for: The AraC Negative Regulator family modulates the activity of histone-like proteins in pathogenic bacteria
Source: PLoS Pathog. 2017 Aug 14;13(8):e1006545. doi: 10.1371/journal.ppat.1006545 (PMC5570504; doi:10.1371/journal.ppat.1006545)
Supplement: S1 Fig — Differentially expressed genes detected by using RNA-seq analysis (p<0.05). Genes for EAEC strain 042 vs 042aar (panel A) or 042aar vs 042aar(pAar) (panel B). AggR-regulated genes are indicated in yellow. (PPTX) [file ppat.1006545.s001.pptx]

## Slide 1
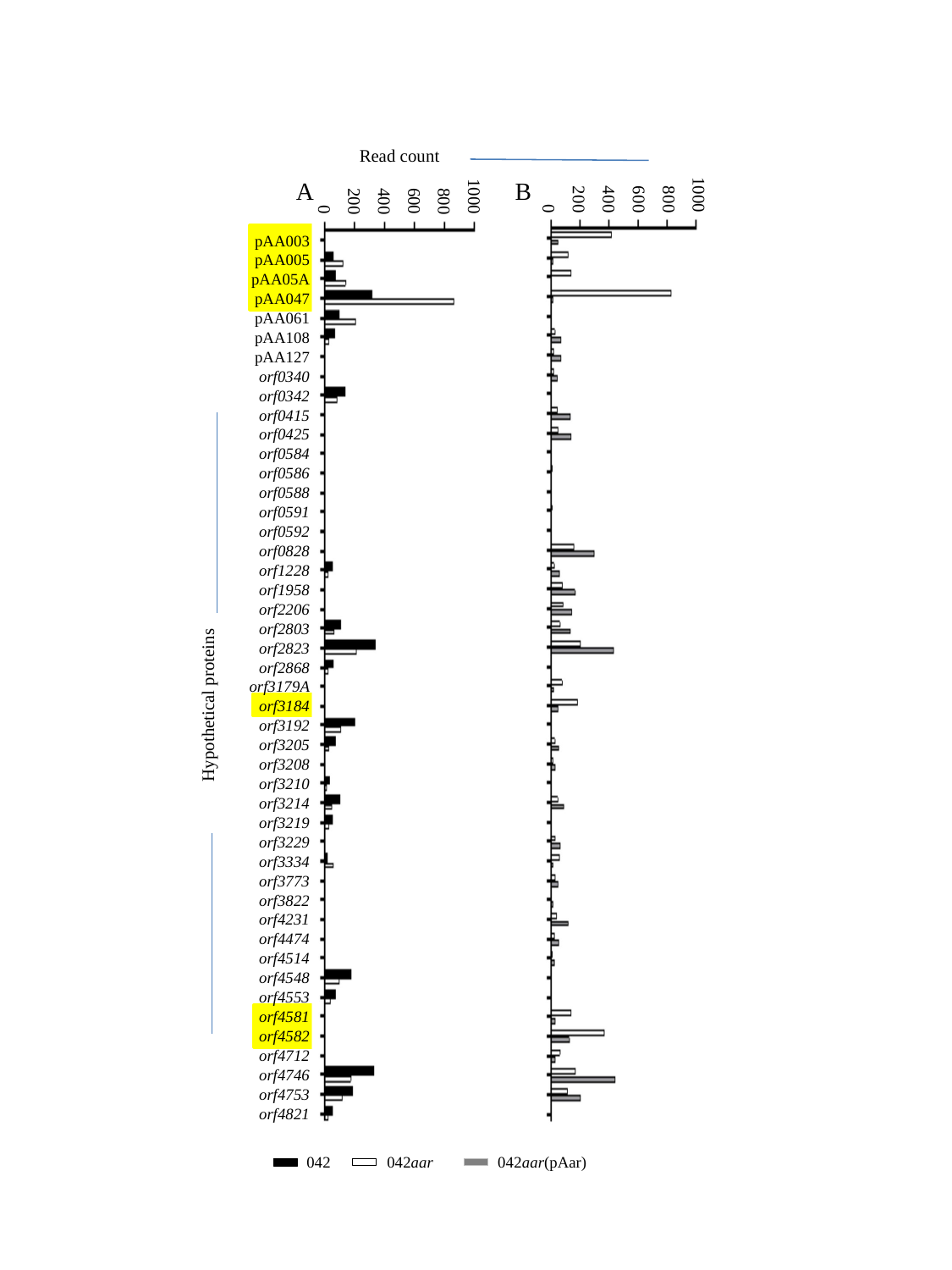

1000
800
600
400
200
0
Read count
A 	 B
1000
800
600
400
200
0
pAA003
pAA005
pAA05A
pAA047
pAA061
pAA108
pAA127
orf0340
orf0342
orf0415
orf0425
orf0584
orf0586
orf0588
orf0591
orf0592
orf0828
orf1228
orf1958
orf2206
orf2803
orf2823
orf2868
orf3179A
orf3184
orf3192
orf3205
orf3208
orf3210
orf3214
orf3219
orf3229
orf3334
orf3773
orf3822
orf4231
orf4474
orf4514
orf4548
orf4553
orf4581
orf4582
orf4712
orf4746
orf4753
orf4821
Hypothetical proteins
042 042aar	 042aar(pAar)
