# Supplementary material for: The AraC Negative Regulator family modulates the activity of histone-like proteins in pathogenic bacteria
Source: PLoS Pathog. 2017 Aug 14;13(8):e1006545. doi: 10.1371/journal.ppat.1006545 (PMC5570504; doi:10.1371/journal.ppat.1006545)
Supplement: S2 Fig — Differentially expressed genes detected by using RNA-seq analysis (p<0.05). EAEC strain 042 vs 042aar (panel A) or 042aar vs 042aar(pAar) (panel B) are showed in the graphs. AggR-regulated genes are indicated in yellow. (PPTX) [file ppat.1006545.s002.pptx]

## Slide 1
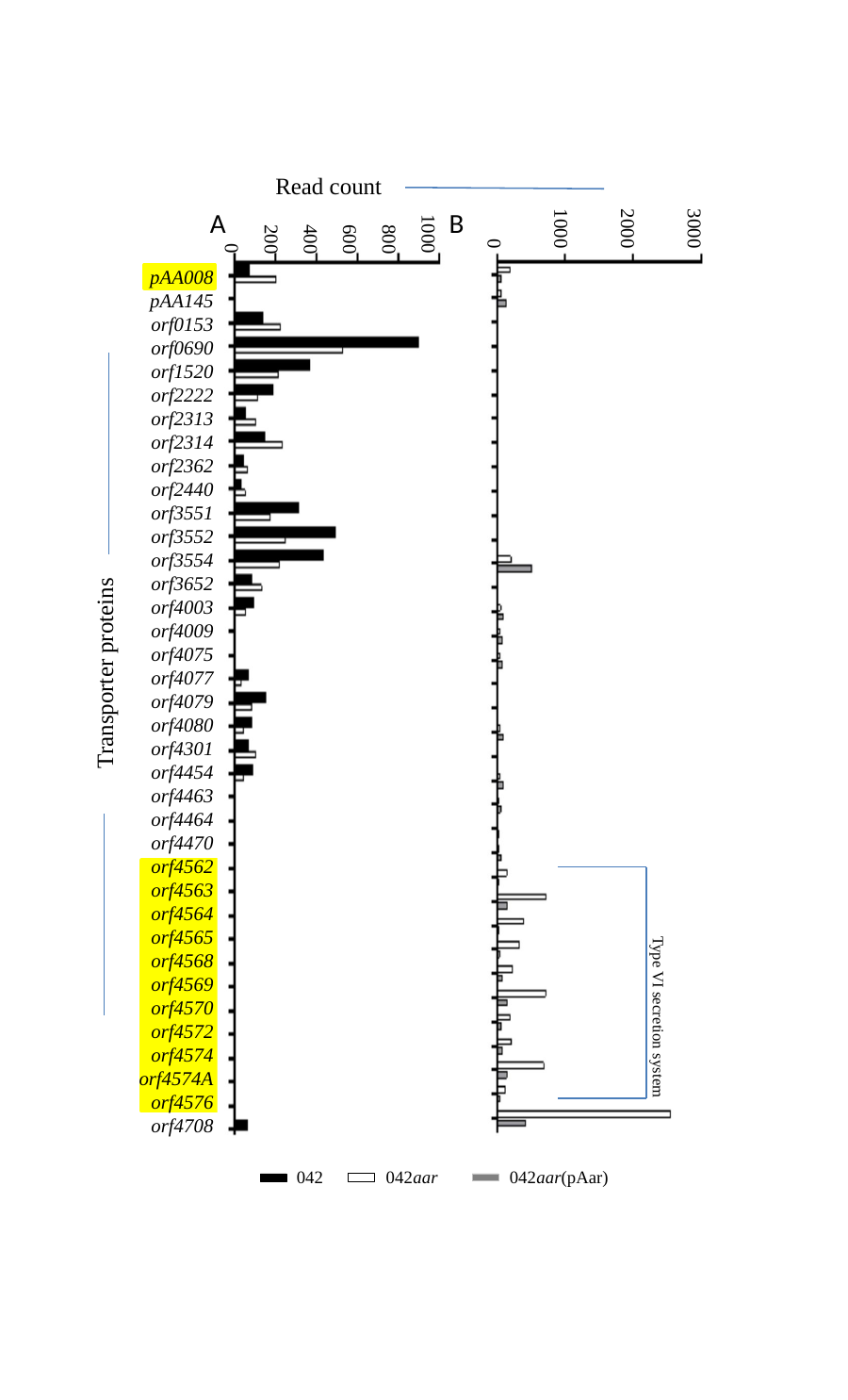

Read count
A 	 B
3000
2000
1000
0
1000
800
600
400
200
0
Type VI secretion system
pAA008
pAA145
orf0153
orf0690
orf1520
orf2222
orf2313
orf2314
orf2362
orf2440
orf3551
orf3552
orf3554
orf3652
orf4003
orf4009
orf4075
orf4077
orf4079
orf4080
orf4301
orf4454
orf4463
orf4464
orf4470
orf4562
orf4563
orf4564
orf4565
orf4568
orf4569
orf4570
orf4572
orf4574
orf4574A
orf4576
orf4708
Transporter proteins
042 042aar	 042aar(pAar)
