# Supplementary material for: The AraC Negative Regulator family modulates the activity of histone-like proteins in pathogenic bacteria
Source: PLoS Pathog. 2017 Aug 14;13(8):e1006545. doi: 10.1371/journal.ppat.1006545 (PMC5570504; doi:10.1371/journal.ppat.1006545)
Supplement: S3 Fig — Differentially expressed genes detected by using RNA-seq analysis (p<0.05). EAEC strain 042 vs 042aar (panel A) or 042aar vs 042aar(pAar) (panel B) are showed in the graphs. (PPTX) [file ppat.1006545.s003.pptx]

## Slide 1
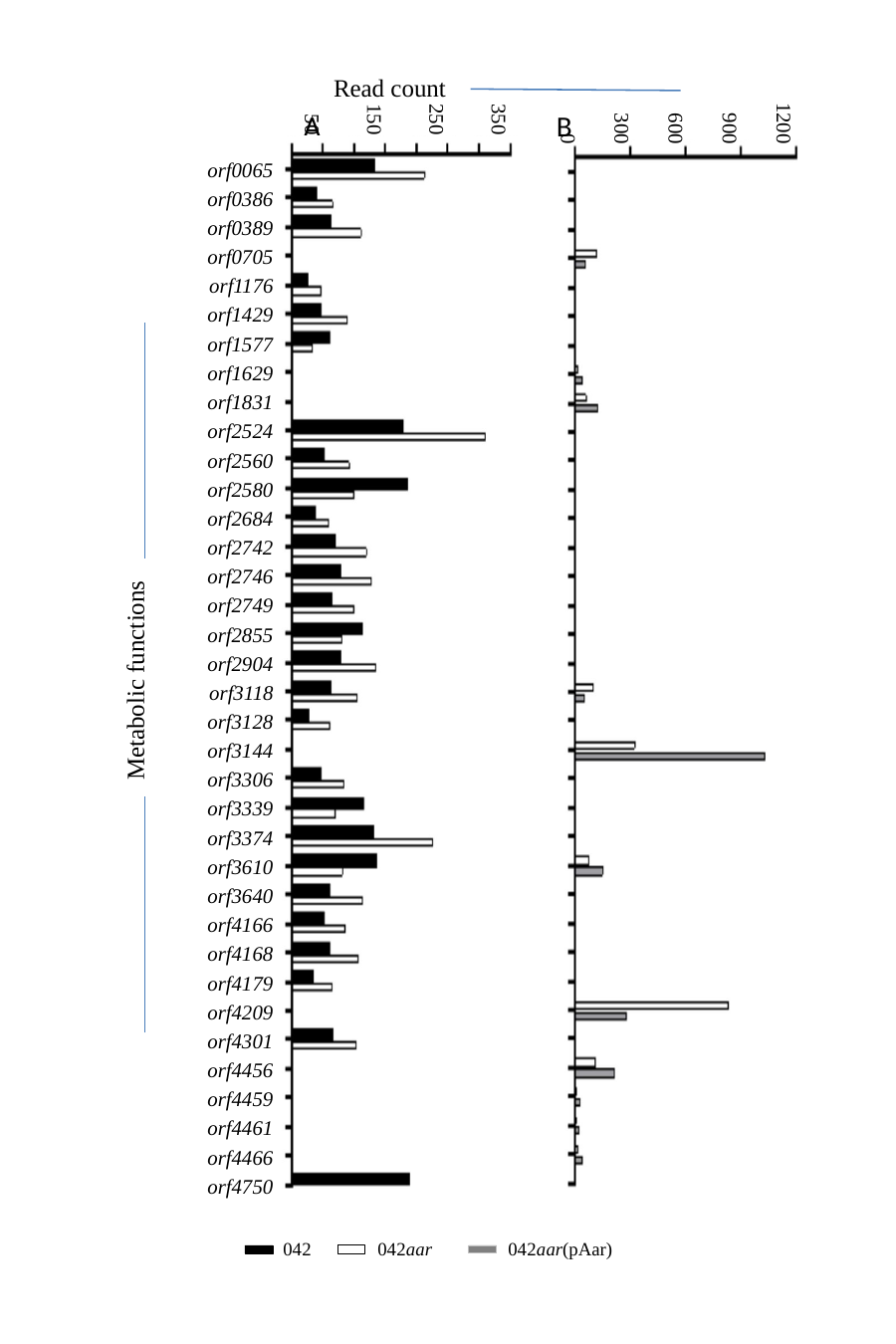

1200
900
600
300
0
350
250
150
50
Read count
A 	 B
orf0065
orf0386
orf0389
orf0705
orf1176
orf1429
orf1577
orf1629
orf1831
orf2524
orf2560
orf2580
orf2684
orf2742
orf2746
orf2749
orf2855
orf2904
orf3118
orf3128
orf3144
orf3306
orf3339
orf3374
orf3610
orf3640
orf4166
orf4168
orf4179
orf4209
orf4301
orf4456
orf4459
orf4461
orf4466
orf4750
Metabolic functions
042 042aar	 042aar(pAar)
