# Supplementary material for: The AraC Negative Regulator family modulates the activity of histone-like proteins in pathogenic bacteria
Source: PLoS Pathog. 2017 Aug 14;13(8):e1006545. doi: 10.1371/journal.ppat.1006545 (PMC5570504; doi:10.1371/journal.ppat.1006545)
Supplement: S4 Fig — Differentially expressed genes detected by using RNA-seq analysis (p<0.05). EAEC strain 042 vs 042aar (panel A) or 042aar vs 042aar(pAar) (panel B) are showed in the graphs. AggR-regulated genes are indicated in yellow. (PPTX) [file ppat.1006545.s004.pptx]

## Slide 1
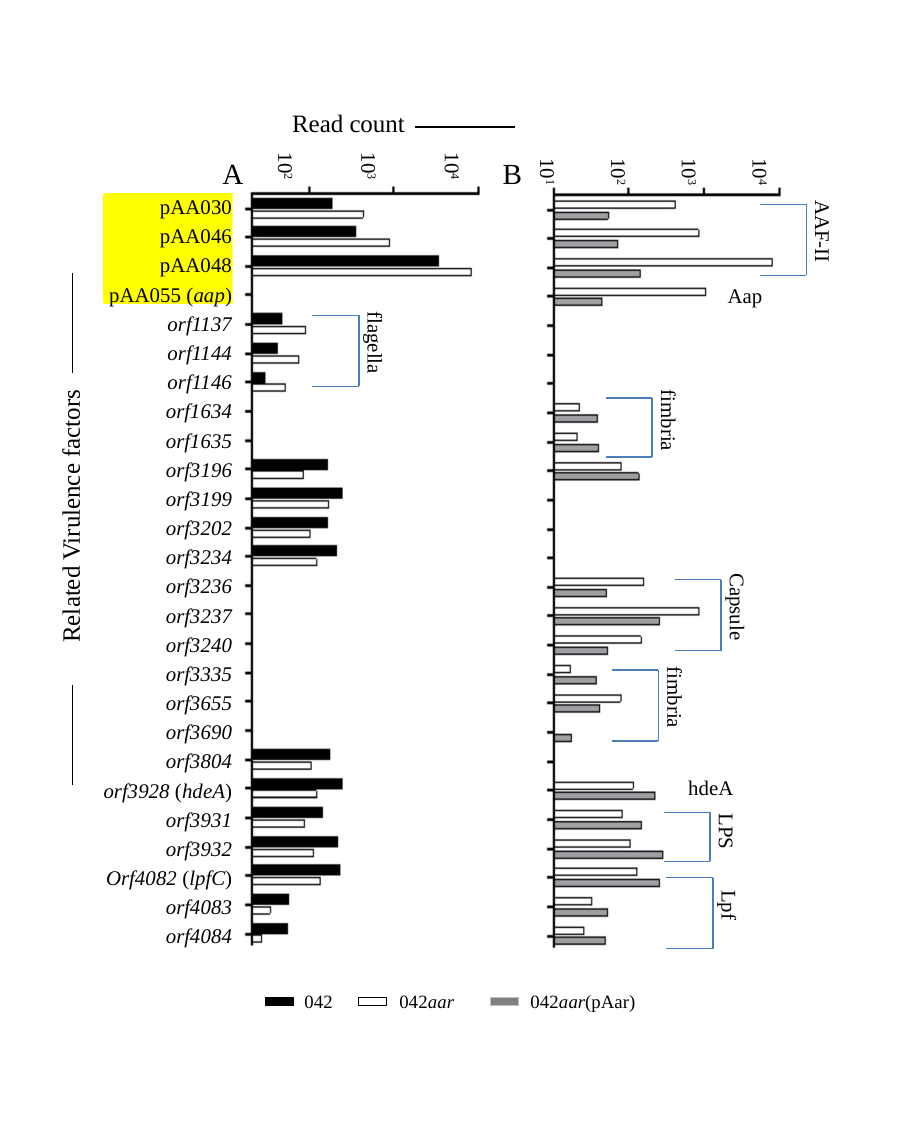

104
103
102
Read count
104
103
102
101
AAF-II
fimbria
Capsule
fimbria
hdeA
LPS
Lpf
A 	 B
pAA030
pAA046
pAA048
pAA055 (aap)
orf1137
orf1144
orf1146
orf1634
orf1635
orf3196
orf3199
orf3202
orf3234
orf3236
orf3237
orf3240
orf3335
orf3655
orf3690
orf3804
orf3928 (hdeA)
orf3931
orf3932
Orf4082 (lpfC)
orf4083
orf4084
Related Virulence factors
Aap
flagella
042 042aar	 042aar(pAar)
