# Supplementary material for: The AraC Negative Regulator family modulates the activity of histone-like proteins in pathogenic bacteria
Source: PLoS Pathog. 2017 Aug 14;13(8):e1006545. doi: 10.1371/journal.ppat.1006545 (PMC5570504; doi:10.1371/journal.ppat.1006545)
Supplement: S5 Fig — Differentially expressed genes detected using RNA-seq analysis (p<0.05). EAEC strain 042 vs 042aar (panel A) or 042aar vs 042aar(pAar) (panel B) are showed in the graphs. AggR is indicated in yellow. (PPTX) [file ppat.1006545.s005.pptx]

## Slide 1
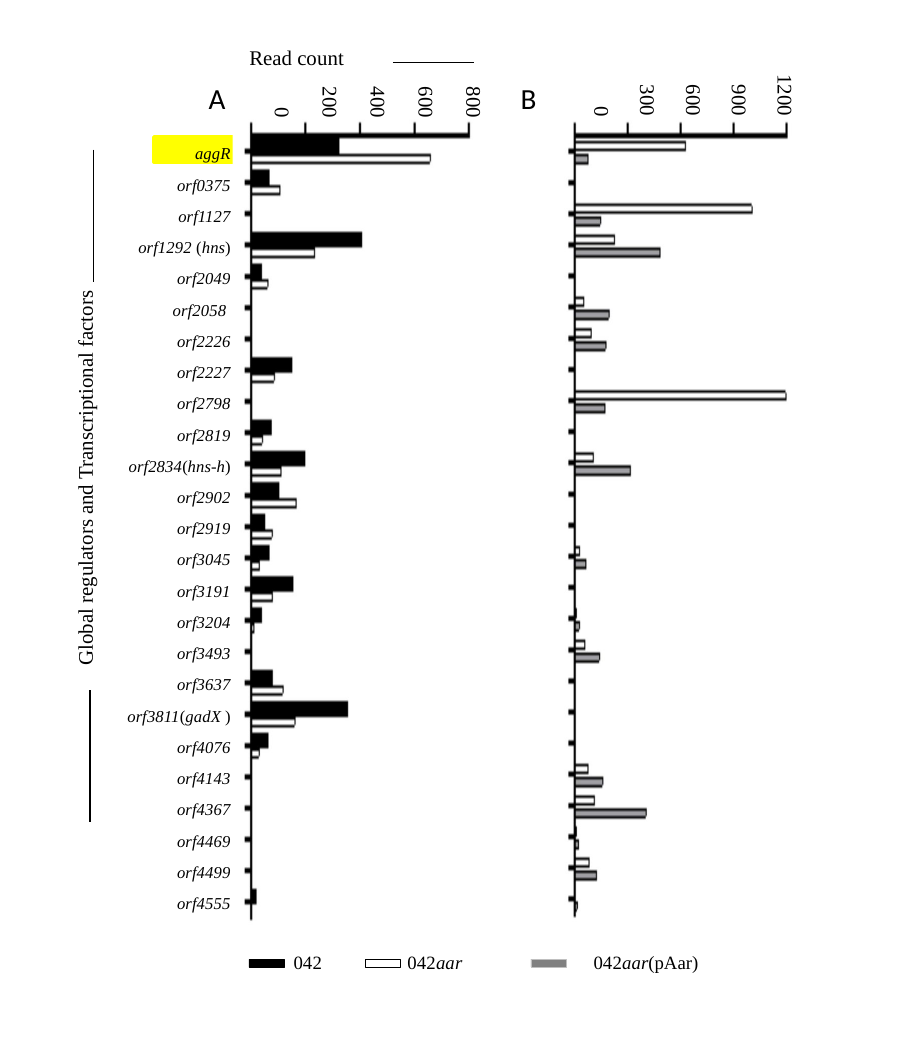

800
600
400
200
0
1200
900
600
300
0
Read count
A 	 B
aggR
orf0375
orf1127
orf1292 (hns)
orf2049
orf2058
orf2226
orf2227
orf2798
orf2819
orf2834(hns-h)
orf2902
orf2919
orf3045
orf3191
orf3204
orf3493
orf3637
orf3811(gadX )
orf4076
orf4143
orf4367
orf4469
orf4499
orf4555
Global regulators and Transcriptional factors
042 042aar	042aar(pAar)
