# Supplementary material for: The AraC Negative Regulator family modulates the activity of histone-like proteins in pathogenic bacteria
Source: PLoS Pathog. 2017 Aug 14;13(8):e1006545. doi: 10.1371/journal.ppat.1006545 (PMC5570504; doi:10.1371/journal.ppat.1006545)
Supplement: S6 Fig — Differentially expressed genes detected by using RNA-seq analysis (p<0.05). EAEC strain 042 vs 042aar (panel A) or 042aar vs 042aar(pAar) (panel B) are showed in the graphs. (PPTX) [file ppat.1006545.s006.pptx]

## Slide 1
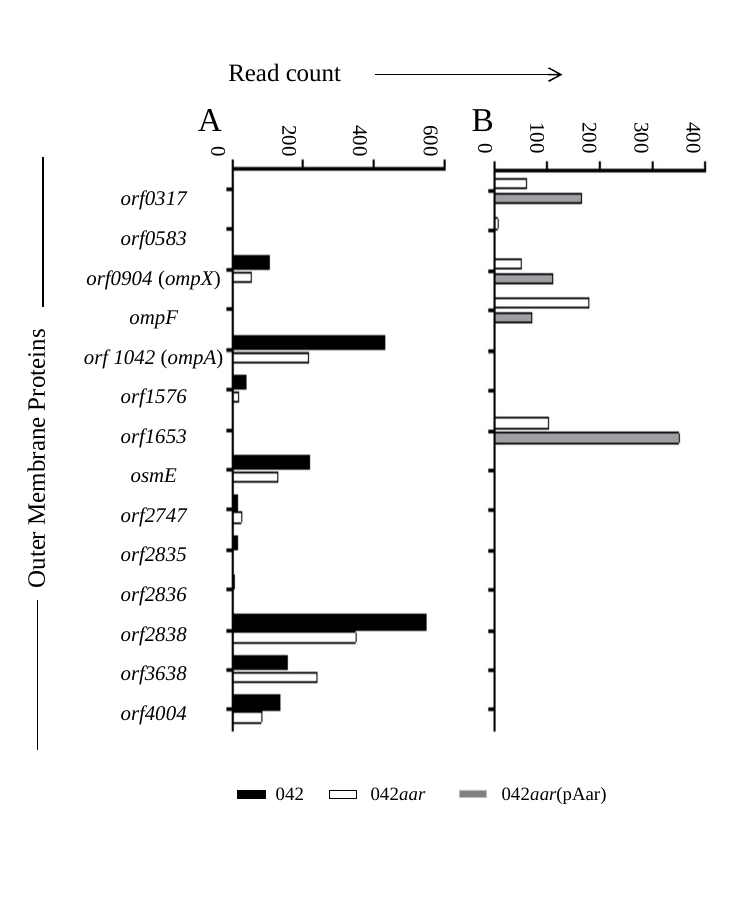

600
400
 200
0
400
300
200
100
0
Read count
A	 B
orf0317
orf0583
orf0904 (ompX)
ompF
orf 1042 (ompA)
orf1576
orf1653
osmE
orf2747
orf2835
orf2836
orf2838
orf3638
orf4004
Outer Membrane Proteins
042 042aar	 042aar(pAar)
