# Supplementary figures and images for: The AraC Negative Regulator family modulates the activity of histone-like proteins in pathogenic bacteria
Source: PLoS Pathog. 2017 Aug 14;13(8):e1006545. doi: 10.1371/journal.ppat.1006545 (PMC5570504; doi:10.1371/journal.ppat.1006545)

## Slide 1
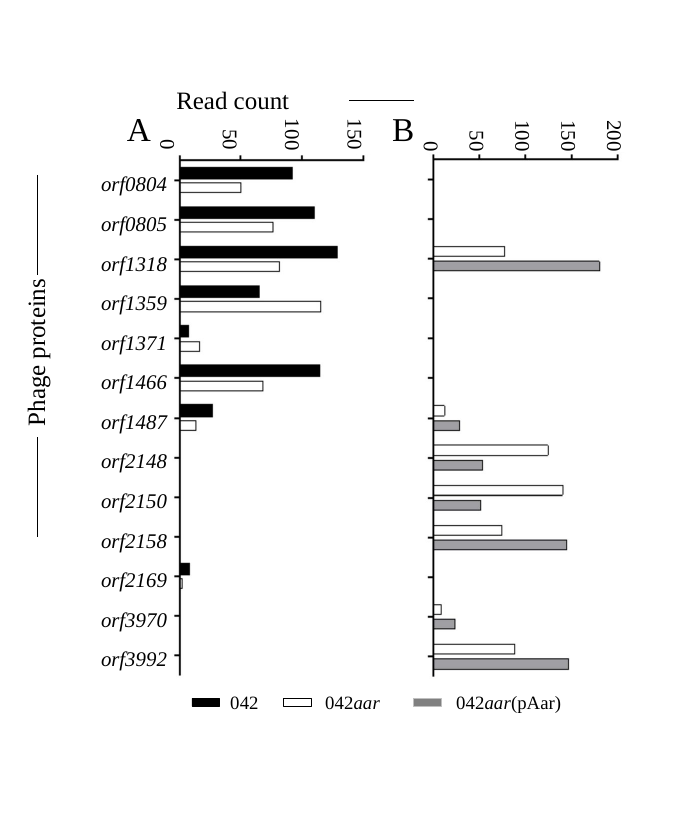

200
150
100
50
0
150
100
50
0
Read count
orf0804
orf0805
orf1318
orf1359
orf1371
orf1466
orf1487
orf2148
orf2150
orf2158
orf2169
orf3970
orf3992
Phage proteins
042 042aar	 042aar(pAar)
A	 B

Supplement: S7 Fig — Differentially expressed genes detected by using RNA-seq analysis (p<0.05). EAEC strain 042 vs 042aar (panel A) or 042aar vs 042aar(pAar) (panel B) are showed in the graphs. (PPTX) [file ppat.1006545.s007.pptx]

## Slide 1
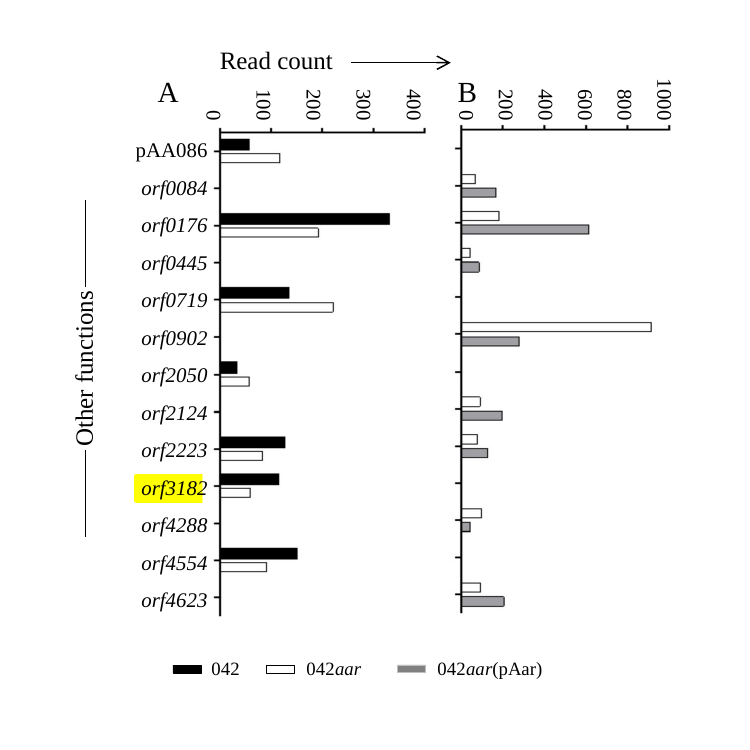

1000
800
600
400
200
0
400
300
200
100
0
Read count
pAA086
orf0084
orf0176
orf0445
orf0719
orf0902
orf2050
orf2124
orf2223
orf3182
orf4288
orf4554
orf4623
Other functions
A		B
042 042aar	 042aar(pAar)

Supplement: S8 Fig — Differentially expressed genes detected by using RNA-seq analysis (p<0.05). EAEC strain 042 vs 042aar (panel A) or 042aar vs 042aar(pAar) (panel B) are showed in the graphs. AggR-regulated gene is indicated in yellow. (PPTX) [file ppat.1006545.s008.pptx]

## Slide 1
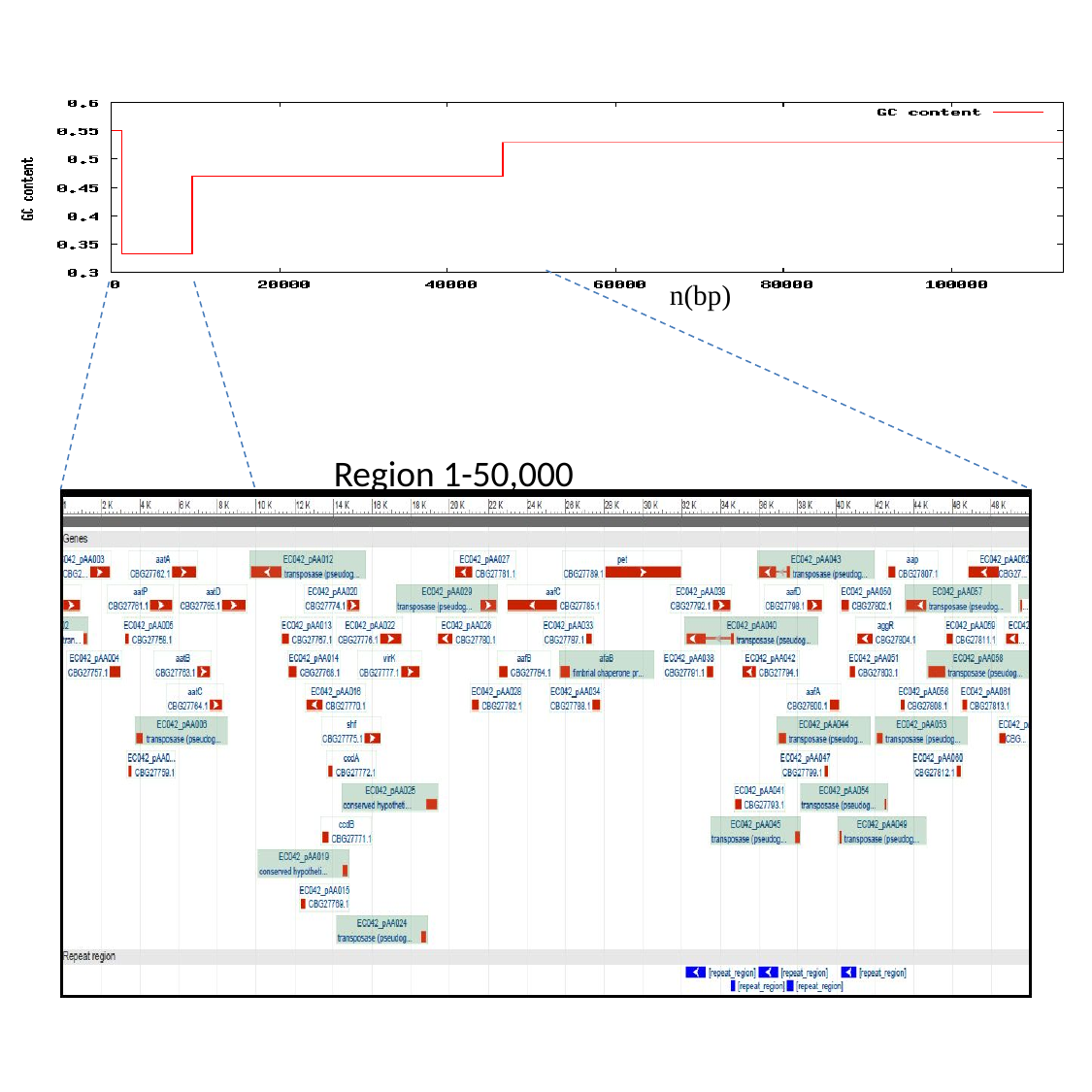

n(bp)
Region 1-50,000

Supplement: S9 Fig — (PPTX) [file ppat.1006545.s009.pptx]

## Slide 1
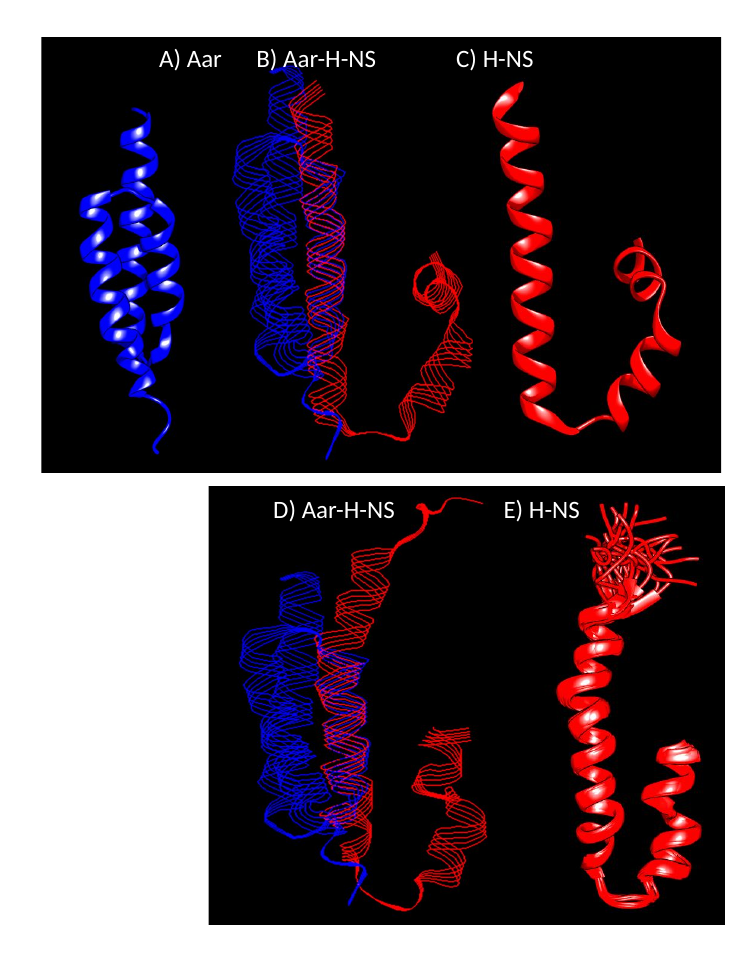

A) Aar B) Aar-H-NS C) H-NS
D) Aar-H-NS E) H-NS

Supplement: S10 Fig — The molecular interaction between Aar (Panel A) and H-NS (Panel C) of E. coli (1N18) (panel B), or Aar and H-NS (Panel E) of S. typhimurium (1LR1) (panel D) was modeled by TM-score, RasMol and the UCSF Chimera package. The second α-helix from Aar was predicted to overlap with the H-NS oligomerization domain. (PPTX) [file ppat.1006545.s010.pptx]

## Slide 1
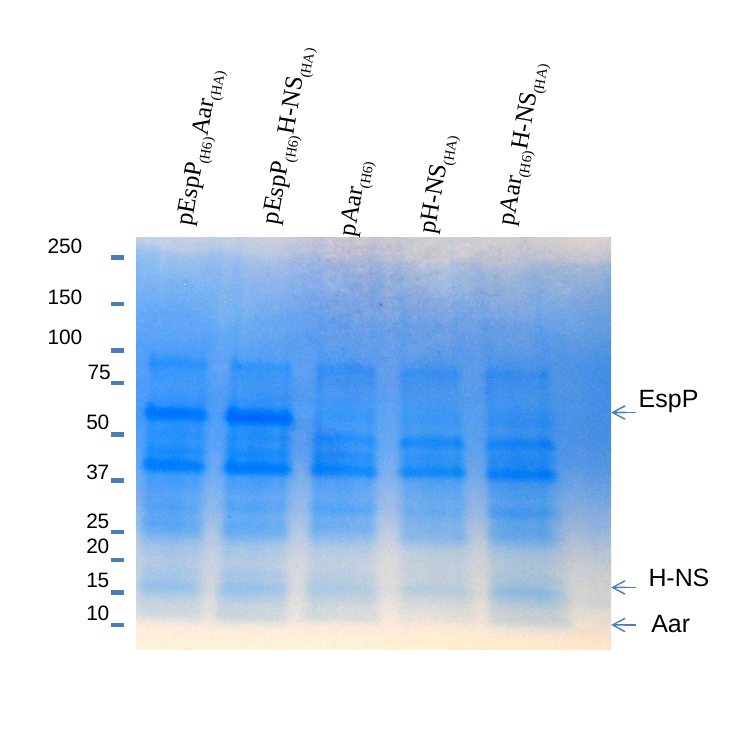

pEspP(H6)H-NS(HA)
pAar(H6)H-NS(HA)
pEspP(H6)Aar(HA)
pH-NS(HA)
pAar(H6)
250
150
100
 75
50
37
25
20
15
10
EspP
H-NS
Aar

Supplement: S11 Fig — E. coli K-12 lysates were analyzed by SDS-PAGE and used in the pull-down assay. (PPTX) [file ppat.1006545.s011.pptx]
