# Supplementary material for: The AraC Negative Regulator family modulates the activity of histone-like proteins in pathogenic bacteria
Source: PLoS Pathog. 2017 Aug 14;13(8):e1006545. doi: 10.1371/journal.ppat.1006545 (PMC5570504; doi:10.1371/journal.ppat.1006545)
Supplement: S1 Table — (DOCX) [file ppat.1006545.s012.docx]

| **Name** | **Characteristics** | **Source** |
| --- | --- | --- |
| **Strains** | | |
| EAEC 042 | Enteroaggregative *E. coli* strain 042, GenBank FN554766 | 49 |
| 042*aggR* | 042 derivative carrying *aggR* deletion | 10,50 |
| 042*aar* | 042 derivative carrying *aar* deletion | 1 |
| 042 pAA(-) | 042 derivative pAA-less | 1 |
| 042*orf1292* | 042 derivative carrying *hns* deletions | This study |
| 042*orf2834* | 042 derivative carrying *hns_(h)_* deletion | This study |
| 042*stpA* | 042 derivative carrying *stpA* deletions | This study |
| 042*aar orf1292* | 042 derivative carrying *aar* and *hns* deletions | This study |
| 042*aar orf2834* | 042 derivative carrying *aar* and *hns_(h)_* deletion | This study |
| 042*orf1292 orf2834* | 042 derivative carrying *hns* and *hns_(h)_* deletion | This study |
| *E. coli* BTH101 | *E. coli* reporter strain for BACTH system | Euromedex |
| *E.coli* K-12 BW25113 | *E. coli* K-12 (*araD-araB*)567∆(*rhaD-rhaB*)568∆l*acZ*4787 *(::rrnB*-3) *hsdR*514 *rph*-1. Keio collection. | 55 |
| *E.coli* K-12 BW25113*hns* | *E. coli* K-12 (*araD-araB*)567∆(*rhaD-rhaB*)568∆*lacZ*4787 (::*rrnB*-3) *hsdR*514 *rph*-1∆*hns.* *hns* gene was deleted by λ-red technology. Keio collection. | 55 |
| **Plasmids** | | |
| pEF-ENTR-lacZ | pEF-ENTR-LacZ (710-1) was a gift from Eric Campeau (Addgene plasmid # 17430) | 54 |
| pP*_H-NS_*LacZ | LacZ reporter plasmid encoding P*_H-NS_*-LacZ fusion | This study |
|  | **pBAD30 derivatives** |  |
| pBAD30 | Cloning and expression vector (Amp^r^) | Lab collection |
| pAar | pBAD30 derivative encoding Aar from EAEC 042 ( also known as pOrf60-2) | 1 |
| pAar_(H6)_H-NS_(HA)_ | pBAD30 derivative encoding Aar and H-NS tagged with H6 and HA respectively | Lab collection |
|  | **pMAL-c5x derivatives** |  |
| pMAL-c5x | Expression vector to generate fusions to the C-terminus of maltose binding protein (MBP) | New England Biolabs |
| pMALAar | pMAL-c5x derivative encoding Aar | 2 |
| pMALHNS | pMAL-c5x derivative encoding HNS (orf1292) | This study |
|  | **Control plasmids for Two-hybrid system assays** |  |
| pUT18 | BACTH vector for fusions to the C-terminus of fragment T18 | Euromedex |
| pKNT25 | BACTH vector for fusions to the C-terminus of fragment T25 | Euromedex |
| pUT18CZip | pUT18C containing Zip protein (positive control BACTH system) | Euromedex |
| pKTZip | pKT25 containing Zip protein (positive control BACTH system) | Euromedex |
| pUT18C | BACTH vector containing fragment T18, used as negative control | Euromedex |
| pKT25 | BACTH vector containing fragment T25, used as negative control | Euromedex |
|  | **pKNT25 derivatives** |  |
| pKNT0808 | Plasmid encoding orf 0808 fused to the T25 fragment of CyaA | This study |
| pKNT1127 | Plasmid encoding orf 1127 fused to the T25 fragment of CyaA | This study |
| pKNT1292 | Plasmid encoding orf 1292 fused to the T25 fragment of CyaA | This study |
| pKNT2020 | Plasmid encoding orf 2020 fused to the T25 fragment of CyaA | This study |
| pKNT2058 | Plasmid encoding orf 2058 fused to the T25 fragment of CyaA | This study |
| pKNT2834 | Plasmid encoding orf 2834 fused to the T25 fragment of CyaA | This study |
| pKNT2881 | Plasmid encoding orf 2881 fused to the T25 fragment of CyaA | This study |
| pKNT2888 | Plasmid encoding orf 2888 fused to the T25 fragment of CyaA | This study |
| pKNT3191 | Plasmid encoding orf 3191 fused to the T25 fragment of CyaA | This study |
| pKNT3204 | Plasmid encoding orf 3204 fused to the T25 fragment of CyaA | This study |
| pKNT4499 | Plasmid encoding orf 4499 fused to the T25 fragment of CyaA | This study |
| pKNT4555 | Plasmid encoding orf 4555 fused to the T25 fragment of CyaA | This study |
|  | **pUT18 derivatives** |  |
| pUT18Aar | Plasmid encoding Aar fused to the T18 fragment of CyaA | 2 |
| pUT18Cnr2 | Plasmid encoding Cnr2 fused to the T18 fragment of CyaA | 2 |
| pUT18ANR*_Vibrio_* | Plasmid encoding ANR*_Vibrio_* fused to the T18 fragment of CyaA | 2 |
