# Supplementary material for: The AraC Negative Regulator family modulates the activity of histone-like proteins in pathogenic bacteria
Source: PLoS Pathog. 2017 Aug 14;13(8):e1006545. doi: 10.1371/journal.ppat.1006545 (PMC5570504; doi:10.1371/journal.ppat.1006545)
Supplement: S2 Table — (DOCX) [file ppat.1006545.s013.docx]

| **Name** | **DNA Sequences (5→3)** |
| --- | --- |
| **RT-PCR primers** | |
| Hypothetical proteins | |
| EC042_1228 sense  EC042_1228 reverse | GCAAGCGTGACCAGACCTAT  CTTGCTCGGTTAATGCCTGT |
| EC042_2823 sense  EC042_2823 reverse | GGAGTCCCAAAATACGCAAA  TATGCAGGGCAAAACATGAA |
| EC042_3192 sense  EC042_3192 reverse | ACACTGCCAGTCCATCCTCT  ATTGTCCGCCAGCACTTATC |
| EC042_3205 sense  EC042_3205 reverse | AACACGTCAGCTCATTCACG  CCAGGTCTGTCTGCTGTTGA |
| EC042_3334 sense  EC042_3334 reverse | TGTTTATTGCCTGGTACTGGA  CACTTCTTTATCGGAGCGTTT |
| EC042_4746 sense  EC042_4746 reverse | AAACATGGTCGCATCAATCA  ACCAAGTTGTCGAGCGTTCT |
| EC042_4753 sense  EC042_4753 reverse | AGATGGCCGTAACCAATCTG  CTGAGCGGTGGGATTAACAT |
| Transporter proteins | |
| EC042_0690 sense  EC042_0690 reverse | TCACTTCCGGCACTACCTCT  CCGTAGGCTTCCTGAGACTG |
| EC042_4080 sense  EC042_4080 reverse | AGCTACCTGGCGAAAGTGAA  GGTGTACGCCAGGTTGTTCT |
| Putative virulence factors | |
| EC042_3928 sense  EC042_3928 reverse | TGGTAGAGGGGATTGGACTG  TCGCTTCAACCCTTGATTTC |
| EC042_3931 sense  EC042_3931 reverse | TGAAGCGCTACAAGCAGAGA  GCTGCTGTTTCGCAATATGA |
| EC042_3932 sense  EC042_3932 reverse | ATAAGTGCGTTGGGCTTACG  ACGCTTAGAACCGCAAAAGA |
| EC042_4082 sense  EC042_4082 reverse | TGCAAAGTCTAACGCCACTG  ACGTTTGAGGATGGCTAACG |
| Transcriptional factors/Global regulators | |
| AggR sense  AggR reverse | CCTAAAGGATGCCCTGATGA  GAATCGTCAGCATCAGCTACA |
| EC042_1292 sense  EC042_1292 reverse | ATAGCCTTGCTGCCGTTAAA  CGAGGGATTTACCTTGCTCA |
| EC042_2834 sense  EC042_2834 reverse | TCAGGCTCGTGAACTGACAC  TGGGCTAACAGTTGAAAGCA |
| EC042_3045 sense  EC042_3045 reverse | CGGTACATAACGCCGATCTT  CGAAATCCGCGTCTGATAAT |
| EC042_3191 sense  EC042_3191 reverse | CTTTTCGCTTGCTGCTTACC  TGTGTTTTCATTCCCAACCA |
| EC042_3204 sense  EC042_3204 reverse | ATCCCCTGATCGACATGAAC  CCTCGTGATTCCTCAATTCG |
| Acid resistance operon in 042 | |
| EC042_3803 sense  EC042_3803 reverse | GCCAATGAATCCGCTAAAGA  TTTTCTGCGGGTTTTTCTTG |
| EC042_3804 sense  EC042_3804 reverse | GAAGATTTCCTGGCTGTGGA  TTGTCCCATTCGCCTTTAAC |
| EC042_3805 sense  EC042_3805 reverse | TTATTCAGCAACCGCAGTCA  CTTTCATTGAACGCTGACGA |
| EC042_3806 sense  EC042_3806 reverse | TGCCCCATAAGAATTCACAA  GTGATACCCAGGGTGACGAT |
| EC042_3810 sense  EC042_3810 reverse | AATTTGGCAGACGATTTTGC  CCCCGGAGTCATTGAAACTA |
| EC042_3811 sense  EC042_3811 reverse | TGCAACATGCTTTGCAACTT  ATTCCCTTGCGCAACAATAC |
| Outer membrane Protein | |
| EC042_0904 sense  EC042_0904 reverse | ACGACTGGGCAAGCATCTAC  ATCCAGGTGCCTACGTCAAC |
| EC042_1042 sense  EC042_1042 reverse | AACGTACCTGGTGGCGTATC  GACCGAAACGGTAGGAAACA |
| EC042_1904 sense  EC042_1904 reverse | CGGGTTGTACGGCTTATGAT  TCACGTTGACCCAGGATGTA |
| Others | |
| Aap sense  Aap reverse | TGGAACGCAGATAATGTGGA  TACCCCAGAGACAGACACCC |
| EC042_2223 sense  EC042_2223 reverse | TAAACTCGACGCGCTGTATG  CTGCGTGAGTAAAACGACCA |
| **Regulatory region for H-NS (orf1292) (1,377,848 – 1,377,154; GenBank FN554766) fused to *lacZ* gene** | |
| pHNSlacZ NheI sense  pHNSlacZ BamHI rev | ATGCTCgctagcTCAGCCACAGGCCCTCAATGATATGAATTAAAGTTGGC  ATGCTCggatccCCAGCATTTCTTCCAGCGTTTCAAGTGTAC |
| **DNA probe for EMSA (ProV region, 3,066,656 – 3,067,066; GenBank FN554766)** | |
| ProV sense  ProV reverse | ATCACGCAAATATTTTGTGGTGATCTACACTGACACTCTGTTGC  TAGCCCAGTTTTTTCCAGAATTTGTTCTTTTGAAAGTCC |
| **DNA probe for EMSA (orf2223, region, 2,310,128 – 2,310,293; GenBank FN554766)** | |
| Orf2223 sense  Orf2223 rev | TTATCAGGTTGCCAGTGTGGTTGGTGGTGGATTTACA  TCAAGCGCGTTGATTGTCTTTCATCAACAAAGCGG |
| **DNA probe for EMSA (orf3928, region, 4,177,012 – 4,177,151; GenBank FN554766)** | |
| 3928-2 sense  3928-2 rev | AACTGAAACATTTGAATGGATAATATACAGTATACATTCTG  CCTCTACCAATGTCAAAATATGCAGTGATAATTGTGGTGGATG |
| **DNA probe for EMSA (orf2834, region, 3,024,825 – 3,024,981; GenBank FN554766)** | |
| Orf2834 sense  Orf2834 rev | AATACAATCGAATACTACATTAATAATCAGGAAGA  AAATTTATTATTTGGGTTTGTGATTATCTTTAGCCG |
| **DNA probe for EMSA (orf1292, region, 1,377,242 – 1,377,539; GenBank FN554766)** | |
| Orf1292 sense  Orf1292 reverse | Gaattctcgtaaacacgactaa  cattgtagtaatctcaaac |
| **Screening primers for lambda red technology** | |
| HNS sense  HNS reverse | CCTTCTGAGCTATCATTACAACTGCCTCGCTTGTTATAAGCGGG  GCGCCGGGTGAAAGCGTACCGATGGTTGGCGTGGTTG |
| StpA sense  StpA reverse | GTCAGGCTTGCGGAATTAGCGAGCAGAGAGCGCCGCTCTG  GATCTGGCTCCATTATCCGTGCTGGAACAGTCAGGTCTTCCGGC |
| 2834 sense  2834 reverse | CATGACAACTACTTTGCATCCAAATTACGTTGTTGTATCAAAGC  GCTGTGTTAATGACGAGATATGCTGATGTAATAGTAGCCACCAG |
| **Two hybrid system constructs** | |
| orf0808 BamHI Sense  orf0808 EcoRI Reverse | ATGCGATCCggATCCCatgcaaactctttctgaacgcctcaagaagaggcgaattgcg  ATGCGATCCgAATTCgcgactttaccgcgtttagttccgtactgtaacc |
| orf1127 BamHI Sense  orf1127 EcoRI Reverse | ATGCGATCCggATCCCatggaaaaaaataatgaagtcattcagactcatccgctcG  ATGCGATCCgAATTCtgacgccgatactcgtttaccgggaaatcaccgg |
| orf2888 BamHI Sense  orf2888 EcoRI Reverse | ATGCGATCCggATCCCatgctgattctgactcgtcgagttggtgagaccc  ATGCGATCCgAATTCtaactggactgctgggatttttcagcctggatacgctgg |
| orf2020 BamHI Sense  orf2020 EcoRI Reverse | ATGCGATCCggATCCCatgaatatgctggaaaaaatccagtctcagctggaacatttgagc  ATGCGATCCgAATTCcgatcgtcacttaaattaagtaactgcttatcaaaacgcg |
| orf2881 BamHI Sense  orf2881 EcoRI Reverse | ATGCGATCCggATCCCatggatagttcgtttacgcccattgaacaaatgctaaaatttcgcgcc  ATGCGATCCgAATTCctcatcgcttcgagaaccacaccgtcttgttccatctggtcgagacggg |
| orf2058 BamHI Sense  orf2058 EcoRI Reverse | ATGCGATCCggATCCCatgcatacctccgagttgctgaaacacatttatgacatcaacttg  ATGCGATCCgAATTCgcccttttcttgcgcagcgcttcttcaggctgattaacatcattcagc |
| orf4499 BamHI Sense  orf4499 EcoRI Reverse | ATGCGATCCggATCCCatgcaacaacctgtagttcgcgttggcgaatggcttgttactccgtcc  ATGCGATCCgAATTCtctgaagcgagaaatttgtcgagataaggtacaacataaggaacagaagtctgg |
| orf1292 BamHI Sense  orf1292 EcoRI Reverse | ATGCGATCCggATCCCATGAGCGAAGCACTTAAAATTCTGAACAACATCCGTACTCTTCGTGCGC  ATGCGATCCgAATTCTGCTTGATCAGGAAATCGTCGAGGGATTTACCTTGCTCATCCATTGC |
| orf2834 BamHI Sense  orf2834 EcoRI Reverse | ATGCGATCCggATCCCatgtctgatgctttaaagatcatcaacaatattcgtactctccgtgctcaggctcg  ATGCGATCCgAATTCttgatctcgaaatcttccagtgtcttaccggcttcgagcgcagcagcgatagcacgagg |
| orf3191 BamHI Sense  orf3191 EcoRI Reverse | ATGCGATCCggATCCCatgctcttcagtatgcgtacacgaattgactatctggcggataaatacag  ATGCGATCCgAATTCagatggtatgccggtactttgcgggttgttcctgtcaggttatcgtgcacc |
| orf4555 BamHI Sense  orf4555 EcoRI Reverse | ATGCGATCCggATCCCatggttgcgcaaaaactggaggctgctggttgctggcgcagagcttctgcccgc  ATGCGATCCgAATTCcagaccggtgtaccttttctgaatacctccccggacggactgg |
| orf3204 BamHI Sense  orf3204 EcoRI Reverse | ATGCGATCCggATCCCatgttgacctcaatgacaggccacgactgcgtgttgctgcgtgccgacg  ATGCGATCCgAATTCgctgctgctcctcgtgattcctcaattcgctgctgcatccactgctccacttc |
| **Λ-red deletion of *hns* (orf1292) (Region 1,376,681 – 1,377,424; GenBank FN554766)** | |
| HNS(orf1292) λred sense | CGGCGCAAATAGGGCTATATGCCGCGTCTTTTCTGGCTAATTTTATGAAAAGATATTTATTGGCGGCACAAAATAAAGAACAATTTTGAATTCCTTACATTCCTGGCTATTGCACAACTGAATTTAAGGCTCTATTATTACCTCAACAAACCACCCCAATATAAGTTTGAGATTACTACAgtgtaggctg gagctgcttc |
| HNS(orf1292) λred reverse | AAGTAACATCCGTATCGGTGTTATCCACGAAACGGCGTTGAGTAATCGACGCCGTTTTTTTATAGCTTATTCTTATTAAATTGTCTTAAACCGGACAATAAAAAATCCCGCCGATGGCGGGATTTTTAAGCAAGTGCAATCTACAAAAGATTATTGCTTGATCAGGAAATCGTCGAGGGAATGGGAATTAGCCATGGTCC |
| **Λ-red deletion of *hns(h)* (orf2834) (Region 3,024,998 – 3,025,605; GenBank FN554766)** | |
| HNSh (Orf2834) λred sense | TAATGTCTATACCGTATAATGTATTGATTTTGTGCACAGGAACGATTAAAACTATGATGTTATTGCTTGTATTCTTTACCACAAACATTAGGGAAATCGCATGTCTGATGCTTTAAAGATCATCAACAATATTCGTACTCTCCGTGCTCAGGCTCGTGAACTGACACTCGAAACCTTGGAgtgtaggctggagctgcttc |
| HNSh (Orf2834) λred reverse | TAATTAAGAACTCTTTTATTGGCATGTTAATCCCTGTAGGTTGGTTCAACAAACCCGCCCAAGAAAAATGAGCGGATTTGGTAGACATATGGTATGAAGATTACTTGATCTCGAAATCTTCCAGTGTCTTACCGGCTTCGAGCGCAGCAGCGATAGCACGAGGAGTTCGGCCCTGACCTGATGGGAATTAGCCATGGTCC |
| **Λ-red deletion of *stpA* (Region 3,058,170 – 3,058,930; GenBank FN554766)** | |
| StpA λred sense | ACTCATCAAAATGGCTAATATATAATCTTGAAATTATTCTGAGAGTTTCAGAAAATAACAGCTGAAATAATCTCGCGCAGGACTGTAAATAGATTAAATTTTGTGGAAATATAATAAGTGATCGCTTACACTACGCGACGAAATACTTTTTTGTTTTGGCGTTAAAAGGTTTTCTTTATTGTGTAGGCTGGAGCTGCTTC |
| StpA λred reverse | TGGGTTATACCGTAAATAGTGGTTTCTTCTTATTAAGCGGCTTTGTTGGTGCCGGGTTACTGTTTGCAGGAATTAGCGGTTTTTGTGGGATGGCAAGGTTGTTAGATAAGATGCCGTGGAACCAACGAGCTTGAGAAGCGACGCCGGACGCGCCATAGCAGCGACATCCGGCCTCAGTAAATGGGAATTAGCCATGGTCC |
| **Purification of H-NS** | |
| HNS1292 MAL SENSE  HNS1292 MAL REV | AGCTGCAGCAT ATG AGCGAAGCACTTAAAATTCTGAACAAC  AGCTGCAGGAA TTC TTA TTG CTT GAT CAG GAA ATC GTC GAG GG |
