# Supplementary material for: The AraC Negative Regulator family modulates the activity of histone-like proteins in pathogenic bacteria
Source: PLoS Pathog. 2017 Aug 14;13(8):e1006545. doi: 10.1371/journal.ppat.1006545 (PMC5570504; doi:10.1371/journal.ppat.1006545)
Supplement: S3 Table — (DOCX) [file ppat.1006545.s014.docx]

|  | | | | | |
| --- | --- | --- | --- | --- | --- |
| **Chromosomal genes** | | **RNA-seq**  **042 vs 042*aar***  **Product** | **042** | **042*aar*** |  |
| **ORF** | **Protein Id** |  | **Read.Count.G1** | **Read.Count.G2** | **p.Value** |
| EC042_3334 | CBG36162.1 | Conserved hypothetical protein | 15.10768939 | 62.19332882 | 0.002240097 |
| EC042_3205 | CBG36033.1 | Conserved hypothetical protein | 69.07425225 | 28.79254554 | 0.004118287 |
| EC042_3204 | CBG36032.1 | Putative regulatory protein | 34.97683877 | 13.37955028 | 0.004409381 |
| EC042_3214 | CBG36042.1 | Conserved hypothetical protein | 100.5769423 | 50.10651852 | 0.004613186 |
| EC042_3191 | CBG36016.1 | Putative DNA-binding protein | 150.684922 | 80.13836802 | 0.005770798 |
| EC042_3811 | CBG36636.1 | AraC-family transcriptional regulator, GadX | 351.4309206 | 162.8798908 | 0.0067777 |
| EC042_2313 | CBG35147.1 | HlyD family secretion protein | 48.66655959 | 103.6921308 | 0.006784222 |
| EC042_3196 | CBG36020.1 | Probable microcin H47 secretion/processing ATP-binding protein | 155.136489 | 83.4966146 | 0.007709591 |
| EC042_0904 | CBG33729.1 | Outer membrane protein X | 102.3919087 | 53.40064187 | 0.008743205 |
| EC042_2580 | CBG35412.1 | Phosphohistidine phosphatase | 183.1667056 | 99.26927516 | 0.009083522 |
| EC042_1042 | CBG33864.1 | Outer membrane protein A | 430.085884 | 217.1494841 | 0.009903027 |
| EC042_2834 | CBG35667.1 | Putative histone-like DNA-binding protein (putative H-NS) | 195.4367826 | 112.7129487 | 0.010997667 |
| EC042_2068 | CBG34894.1 | Ferritin-1 | 58.40891706 | 25.84740029 | 0.011029289 |
| EC042_3192 | CBG36017.1 | Conserved hypothetical protein | 201.3733221 | 110.5726316 | 0.011347246 |
| EC042_3554 | CBG36379.1 | ABC transporter, ATP-binding protein | 427.6528069 | 217.6053342 | 0.011635521 |
| EC042_4746 | CBG37565.1 | Conserved hypothetical protein | 326.4594293 | 178.5814405 | 0.011700986 |
| EC042_3928 | CBG36751.1 | Putative lipopolysaccharide biosynthesis protein | 232.2830283 | 120.0098388 | 0.012427187 |
| EC042_1137 | CBG33956.1 | Flagella synthesis protein FlgN | 44.40009045 | 90.03841282 | 0.01308786 |
| EC042_2215 | CBG35041.1 | Yersiniabactin siderophore biosynthetic protein | 230.0417503 | 116.1529932 | 0.013193501 |
| EC042_4084 | CBG36909.1 | Putative major fimbrial subunit (LpfA) | 52.26215283 | 26.73772615 | 0.013656645 |
| EC042_3128 | CBG35953.1 | Methylmalonyl-CoA decarboxylase | 26.54356879 | 60.57997271 | 0.01373002 |
| EC042_3045 | CBG35872.1 | Putative transcriptional regulator | 64.07157855 | 32.97699476 | 0.014520146 |
| EC042_4082 | CBG36907.1 | Fimbrial outer membrane usher protein (LpfC) | 219.9568355 | 134.4720846 | 0.014697632 |
| EC042_2904 | CBG35735.1 | Nitric oxide reductase FlRd-NAD(+) reductase | 76.91453841 | 134.6106065 | 0.016745003 |
| EC042_3932 | CBG36755.1 | Lipid A-core:surface polymer ligase | 203.1882885 | 110.8788609 | 0.017032484 |
| EC042_1490 | CBG34314.1 | ParB-like nuclease | 43.70743833 | 17.91297759 | 0.017100107 |
| EC042_2803 | CBG35636.1 | Putative exported protein | 104.9757441 | 65.15984849 | 0.017114098 |
| EC042_4168 | CBG36995.1 | dTDP-4-oxo-6-deoxy-D-glucose transaminase | 60.3436527 | 107.3352322 | 0.017214478 |
| EC042_3640 | CBG36465.1 | Phosphotriesterase-like protein | 60.1242152 | 114.0517254 | 0.017307943 |
| EC042_1292 | CBG34112.1 | DNA-binding protein (histone-like protein Hlp-II), H-NS | 404.8262759 | 237.9258089 | 0.017579942 |
| EC042_1904 | CBG34730.1 | Osmotically inducible lipoprotein E, OsmE | 215.1518231 | 128.9628338 | 0.017813172 |
| EC042_4554 | CBG37376.1 | ParB-like nuclease | 149.9034897 | 92.27861435 | 0.018020224 |
| EC042_0176 | CBG33008.1 | Chaperone protein, Skp | 330.7945777 | 193.1254843 | 0.018892901 |
| EC042_2440 | CBG35273.1 | Heme exporter protein C | 27.57710292 | 54.25520647 | 0.019683631 |
| EC042_1429 | CBG34252.1 | Gamma-glutamylputrescine oxidoreductase | 45.73095415 | 89.18014857 | 0.019802073 |
| EC042_3552 | CBG36377.1 | ABC transporter, permease protein | 490.5526566 | 248.0358982 | 0.021718811 |
| EC042_1144 | CBG33963.1 | Flagellar basal-body rod protein FlgF | 39.96862436 | 74.23369065 | 0.021861088 |
| EC042_0804 | CBG33628.1 | Prophage protein | 91.22990738 | 50.48755823 | 0.022262859 |
| EC042_4003 | CBG36829.1 | Sugar efflux transporter C | 89.66871808 | 54.72544347 | 0.02281254 |
| EC042_2222 | CBG35048.1 | Shikimate transporter | 186.8494037 | 114.7463936 | 0.023291476 |
| EC042_0389 | CBG33222.1 | 4-hydroxy-2-oxovalerate aldolase | 61.32274628 | 111.1386417 | 0.024327655 |
| EC042_3306 | CBG36134.1 | 1-acyl-glycerol-3-phosphate acyltransferase | 45.09274257 | 84.64672126 | 0.024373491 |
| EC042_1228 | CBG34048.1 | Conserved hypothetical protein | 50.22774889 | 24.1843078 | 0.025781403 |
| EC042_1577 | CBG34402.1 | Putative acetyltransferase | 58.73891096 | 32.97699476 | 0.025904944 |
| EC042_1176 | CBG33994.1 | Thiamine kinase | 23.68418011 | 47.30729447 | 0.027200091 |
| EC042_2362 | CBG35196.1 | ABC transporter, ATP-binding protein | 39.99040058 | 67.10409618 | 0.027207852 |
| EC042_3804 | CBG36629.1 | Putative periplasmic acid stress chaperone, hdeA | 164.835294 | 105.2023145 | 0.027382888 |
| EC042_4548 | CBG37370.1 | Conserved hypothetical protein | 176.1715051 | 100.8682444 | 0.028153897 |
| EC042_4076 | CBG36901.1 | Phosphate transport system regulatory protein, PhoU | 59.60744814 | 31.69494198 | 0.028860569 |
| EC042_1359 | CBG34181.1 | Phage portal protein | 64.61012184 | 115.7682539 | 0.029016436 |
| EC042_3182 | CBG36008.1 | ParB-like nuclease | 113.0572439 | 60.7937168 | 0.029351175 |
| EC042_2838 | CBG35671.1 | Putative membrane protein | 547.3518059 | 350.0156119 | 0.029721707 |
| EC042_4077 | CBG36902.1 | Phosphate ABC transporter, ATP-binding protein | 64.01713801 | 35.02112694 | 0.030167537 |
| EC042_3234 | CBG36063.1 | Capsule polysaccharide export protein | 202.1656425 | 119.6501735 | 0.030198231 |
| EC042_0153 | CBG32985.1 | Ferrichrome transport system permease protein | 133.2563872 | 222.5514511 | 0.031522585 |
| EC042_3374 | CBG36202.1 | 2,4-dienoyl-CoA reductase [NADPH] | 128.8031449 | 225.6569044 | 0.031708256 |
| EC042_2746 | CBG35579.1 | 3-phenylpropionate dioxygenase ferredoxin--NAD(+) reductase | 77.28808474 | 127.6520073 | 0.031976314 |
| EC042_2742 | CBG35575.1 | 3-phenylpropionate dioxygenase alpha subunit | 68.55748519 | 120.3337428 | 0.032643777 |
| EC042_4464 | CBG37286.1 | Phosphonates transport ATP-binding protein | 25.3341496 | 11.68439619 | 0.032747036 |
| EC042_4079 | CBG36904.1 | Phosphate ABC transporter, permease protein | 147.6060958 | 85.40181313 | 0.032762797 |
| EC042_2684 | CBG35517.1 | Hydrogenase-4 component C | 35.87804028 | 59.65758523 | 0.033708493 |
| EC042_2217 | CBG35043.1 | Yersiniabactin siderophore biosynthetic protein | 221.0464855 | 124.9029314 | 0.034482946 |
| EC042_4753 | CBG37572.1 | Conserved hypothetical protein | 184.2982327 | 118.9201557 | 0.034727702 |
| EC042_1466 | CBG34289.1 | Putative phage Protein | 113.9475573 | 68.38984861 | 0.034740062 |
| EC042_2227 | CBG35053.1 | Nitrogen assimilation regulatory protein | 148.5290736 | 86.6731787 | 0.034753386 |
| EC042_4004 | CBG36830.1 | Putative membrane protein | 131.2328714 | 83.45386578 | 0.034861375 |
| EC042_2747 | CBG35580.1 | Putative membrane protein | 11.82031384 | 26.53466927 | 0.034951868 |
| EC042_3199 | CBG36023.1 | Putative microcin H47 biosynthesis protein | 235.1080774 | 164.1977048 | 0.035039175 |
| EC042_3652 | CBG36477.1 | Protein transport protein | 80.56457219 | 133.5209234 | 0.035213861 |
| EC042_1520 | CBG34344.1 | Iron ABC transporter, ATP-binding protein | 363.9020095 | 212.9222861 | 0.035669733 |
| EC042_2868 | CBG35700.1 | Conserved hypothetical protein | 54.80243572 | 26.21775279 | 0.037187329 |
| EC042_4179 | CBG37006.1 | Uroporphyrinogen III synthase | 33.93241653 | 65.59062456 | 0.037341461 |
| EC042_4750 | CBG37569.1 | PTS system EIIC component | 187.1232818 | 111.281275 | 0.037696966 |
| EC042_3638 | CBG36463.1 | Putative membrane protein | 151.6187878 | 239.8482705 | 0.037787054 |
| EC042_2902 | CBG35733.1 | Anaerobic nitric oxide reductase transcription regulator | 99.64307647 | 167.9045177 | 0.037975813 |
| EC042_3551 | CBG36376.1 | Putative ABC transporter, substrate-binding protein | 309.12135 | 176.5549831 | 0.038575682 |
| EC042_4553 | CBG37375.1 | Conserved hypothetical protein | 69.60190743 | 40.0531532 | 0.038710191 |
| EC042_3118 | CBG35943.1 | 2-octaprenyl-6-methoxyphenol hydroxylase | 60.784203 | 105.3338488 | 0.039422448 |
| EC042_1146 | CBG33965.1 | Flagellar L-ring protein | 27.90709681 | 51.37418444 | 0.039987003 |
| EC042_3931 | CBG36754.1 | Lipopolysaccharide heptosyltransferase 1 | 138.3696173 | 85.45524915 | 0.04067429 |
| EC042_4821 | CBG37645.1 | Putative exported protein | 47.99568369 | 24.1736206 | 0.040707681 |
| EC042_1318 | CBG34139.1 | Putative phage protein | 128.0996047 | 81.74802448 | 0.040910511 |
| EC042_2835 | CBG35668.1 | Putative membrane protein | 12.50207785 | 4.173762009 | 0.041022155 |
| EC042_3610 | CBG36435.1 | FKBP-type peptidyl-prolyl cis-trans isomerase | 134.3678134 | 81.66252684 | 0.041183774 |
| EC042_2823 | CBG35656.1 | Hypothetical protein | 335.7411355 | 215.6787613 | 0.041589128 |
| EC042_2223 | CBG35049.1 | AMP nucleosidase | 126.6364083 | 83.05145167 | 0.042641077 |
| EC042_0719 | CBG33542.1 | Two-component sensor kinase | 133.7731543 | 221.0166051 | 0.042809806 |
| EC042_4708 | CBG37528.1 | ABC transporter, permease protein | 59.28834235 | 94.8780694 | 0.042913961 |
| EC042_2836 | CBG35669.1 | Putative membrane protein | 3.254711229 | 0 | 0.043161846 |
| EC042_2560 | CBG35392.1 | Putative semialdehyde dehydrogenase | 51.05273364 | 92.15735544 | 0.043462561 |
| EC042_4301 | CBG37126.1 | Glycerol uptake facilitator protein | 63.92835782 | 103.6386947 | 0.043490985 |
| EC042_0386 | CBG33219.1 | 2-hydroxy-6-ketonona-2,4-dienedioic acid hydrolase | 39.07831096 | 65.88616663 | 0.043714475 |
| EC042_1371 | CBG34193.1 | Phage minor tail protein | 6.850304464 | 16.56680158 | 0.043863027 |
| EC042_2524 | CBG35356.1 | NADH-quinone oxidoreductase subunit G | 177.3047075 | 309.6697927 | 0.044221444 |
| EC042_3202 | CBG36030.1 | Microcin activation protein | 153.9379579 | 100.9964909 | 0.044833817 |
| EC042_2050 | CBG34876.1 | Chemotaxis protein methyltransferase | 32.57977661 | 58.06930318 | 0.045200051 |
| EC042_2819 | CBG35652.1 | SsrA-binding protein | 71.40598576 | 42.1079726 | 0.045821526 |
| EC042_3637 | CBG36462.1 | Putative DNA-binding protein | 76.38688324 | 119.293796 | 0.046187662 |
| EC042_0065 | CBG32899.1 | L-arabinose isomerase | 132.0360799 | 212.6370194 | 0.046200561 |
| EC042_0375 | CBG33208.1 | LysR-family transcriptional regulator | 64.59923373 | 109.963461 | 0.046307857 |
| EC042_4555 | CBG37377.1 | Putative transcriptional regulator | 15.43768329 | 4.999964648 | 0.04631183 |
| EC042_4166 | CBG36993.1 | Glucose-1-phosphate thymidylyltransferase | 50.03008762 | 85.44086229 | 0.046786389 |
| EC042_1576 | CBG34401.1 | Putative membrane protein | 34.92239823 | 18.34745331 | 0.046983094 |
| EC042_2855 | CBG35687.1 | Alpha-amylase | 111.9458177 | 80.23455286 | 0.047009043 |
| EC042_2314 | CBG35148.1 | Multidrug resistance protein | 146.4728934 | 236.3334155 | 0.0470114 |
| EC042_2749 | CBG35582.1 | Putative zinc-binding dehydrogenase | 62.86215937 | 99.42218391 | 0.047197752 |
| EC042_0805 | CBG33629.1 | Prophage protein | 109.3510943 | 76.44183056 | 0.047278782 |
| EC042_2919 | CBG35749.1 | Pormate hydrogenlyase regulatory protein | 46.28038554 | 78.80986677 | 0.047726868 |
| EC042_4083 | CBG36908.1 | Fimbrial chaperone protein, LpfB | 54.16422414 | 33.41147048 | 0.048299787 |
| EC042_3219 | CBG36047.1 | Conserved hypothetical protein | 47.95213125 | 28.37944422 | 0.048691317 |
| EC042_2169 | CBG34995.1 | Putative prophage regulatory protein | 7.872950485 | 2.086881005 | 0.04886066 |
| EC042_2049 | CBG34875.1 | Chemotaxis response regulator protein-glutamate methylesterase | 35.99948479 | 62.20031637 | 0.04913565 |
| EC042_3210 | CBG36038.1 | Hypothetical protein | 29.65505928 | 16.29263392 | 0.049194189 |
| EC042_4080 | CBG36905.1 | Phosphate ABC transporter, substrate-binding protein | 77.65074297 | 46.29242181 | 0.049195955 |
| EC042_4454 | CBG37276.1 | Multidrug resistance protein | 85.18281145 | 43.25109171 | 0.049373694 |
| EC042_0690 | CBG33516.1 | Glutamate/aspartate ABC transporter, substrate-binding protein | 897.3044553 | 529.533415 | 0.049596647 |
| EC042_1487 | CBG34311.1 | Phage lysozome | 26.38945995 | 13.35817587 | 0.049661424 |
| EC042_0342 | CBG33176.1 | Conserved hypothetical protein | 133.9925918 | 85.48731076 | 0.049725622 |
| EC042_2850 | CBG35684.1 | Adhesin autotransporter | 523.5244051 | 350.2684051 | 0.049987665 |
| EC042_3339 | CBG36167.1 | 3,4-dihydroxy-2-butanone 4-phosphate synthase | 113.8713406 | 70.42329359 | 0.050905594 |
