# Supplementary material for: The AraC Negative Regulator family modulates the activity of histone-like proteins in pathogenic bacteria
Source: PLoS Pathog. 2017 Aug 14;13(8):e1006545. doi: 10.1371/journal.ppat.1006545 (PMC5570504; doi:10.1371/journal.ppat.1006545)
Supplement: S4 Table — (DOCX) [file ppat.1006545.s015.docx]

|  | | | | | |
| --- | --- | --- | --- | --- | --- |
| **Chromosomal genes** | | **RNA-seq**  **042*aar* vs 042*aar*(pAar)**  **Product** | **042*aar*** | **042*aar*(pAar)** |  |
| **ORF** | **Protein Id** |  | **Read.Count.G2** | **Read.Count.G3** | **p.Value** |
| EC042_4564 | CBG37385.1 | putative type VI secretion protein | 391.1526515 | 33.66637429 | 2.84E-09 |
| EC042_4565 | CBG37386.1 | putative type VI secretion protein | 328.8831097 | 44.84295032 | 3.71E-07 |
| EC042_2068 | CBG34894.1 | ferritin-1 | 24.80949768 | 138.6134632 | 2.58E-06 |
| EC042_2058 | CBG34884.1 | Transcriptional activator FlhD | 52.3629792 | 195.4933017 | 0.000161537 |
| EC042_4562 | CBG37383.1 | putative type VI secretion protein | 146.7531384 | 29.01084066 | 0.00017458 |
| EC042_4581 | CBG37404.1 | conserved hypothetical protein | 139.4758541 | 32.25928815 | 0.000204336 |
| EC042_4367 | CBG37191.1 | DNA-binding protein HU-alpha | 111.313947 | 406.754509 | 0.000303222 |
| EC042_1127 | CBG33946.1 | biofilm regulator, BssS | 1005.290513 | 147.3858056 | 0.000521018 |
| EC042_2834 | CBG35667.1 | putative histone-like DNA-binding protein, Putative H-NS | 108.2739435 | 317.5990949 | 0.000966259 |
| EC042_2798 | CBG35631.1 | ribosome-associated inhibitor A | 1197.919086 | 176.0670098 | 0.00096724 |
| EC042_0415 | CBG33248.1 | conserved hypothetical protein | 45.19933799 | 136.3058192 | 0.001024205 |
| EC042_1653 | CBG34475.1 | putative membrane protein | 104.5948663 | 351.2654692 | 0.001575558 |
| EC042_4231 | CBG37058.1 | conserved hypothetical protein | 41.60113087 | 118.6725939 | 0.001781815 |
| EC042_3184 | CBG36010.1 | conserved hypothetical protein | 184.9373762 | 51.36399271 | 0.001862684 |
| EC042_3932 | CBG36755.1 | lipid A-core:surface polymer ligase | 106.4180184 | 290.0037678 | 0.002336496 |
| EC042_4563 | CBG37384.1 | putative type VI secretion protein | 725.0278839 | 151.3339607 | 0.002387 |
| EC042_4574 | CBG37394.1 | putative type VI secretion protein | 684.0690142 | 146.2121444 | 0.00240238 |
| EC042_0425 | CBG33258.1 | conserved hypothetical protein | 51.59068846 | 138.6859054 | 0.002502926 |
| EC042_3179A | CBG36005.1 | conserved hypothetical protein | 78.05947316 | 19.1127537 | 0.002628372 |
| EC042_0586 | CBG33413.1 | hypothetical protein | 10.46582874 | 0 | 0.002668336 |
| EC042_0591 | CBG33418.1 | hypothetical protein | 8.001477976 | 0 | 0.002921621 |
| EC042_0176 | CBG33008.1 | chaperone protein, skp | 185.4300339 | 616.9300283 | 0.003169608 |
| EC042_4569 | CBG37389.1 | putative type VI secretion protein | 713.2315607 | 150.9481694 | 0.003302004 |
| EC042_3334 | CBG36162.1 | conserved hypothetical protein | 59.739645 | 12.80079977 | 0.003380984 |
| EC042_0317 | CBG33150.1 | putative membrane protein | 61.07120189 | 166.1047189 | 0.003424672 |
| EC042_2803 | CBG35636.1 | putative exported protein | 62.61472089 | 134.5208018 | 0.004918767 |
| EC042_4574A | CBG37395.1 | putative type VI secretion protein | 125.5592968 | 40.18741669 | 0.005078659 |
| EC042_4464 | CBG37286.1 | phosphonates transport ATP-binding protein | 11.22173297 | 34.26949227 | 0.005451265 |
| EC042_1318 | CBG34139.1 | putative phage protein | 78.48658479 | 180.6100446 | 0.006010148 |
| EC042_4746 | CBG37565.1 | conserved hypothetical protein | 171.4970901 | 446.2180706 | 0.006925956 |
| EC042_4570 | CBG37390.1 | putative type VI secretion protein | 194.2857351 | 63.74661556 | 0.007113364 |
| EC042_4082 | CBG36907.1 | fimbrial outer membrane usher protein, lpfC | 129.205601 | 261.1136642 | 0.007882122 |
| EC042_0583 | CBG33410.1 | putative membrane protein | 7.639912367 | 0 | 0.008803023 |
| EC042_1228 | CBG34048.1 | conserved hypothetical protein | 23.21575668 | 60.33737443 | 0.008832763 |
| EC042_4568 | CBG37388.1 | putative type VI secretion protein | 231.4672086 | 74.81069278 | 0.009133796 |
| EC042_4572 | CBG37392.1 | putative type VI secretion protein | 205.1961245 | 71.01547128 | 0.009639435 |
| EC042_0904 | CBG33729.1 | outer membrane protein X, OmpX | 51.27828237 | 112.6902764 | 0.010353632 |
| EC042_1958 | CBG34784.1 | conserved hypothetical protein | 78.93008293 | 167.5840581 | 0.011137646 |
| EC042_3240 | CBG36069.1 | polysialic acid transport permease protein | 145.9982967 | 52.42534417 | 0.01117942 |
| EC042_2124 | CBG34949.1 | two-component sensor kinase | 94.07881562 | 200.4625625 | 0.011232008 |
| EC042_3236 | CBG36065.1 | putative capsule O-acetyl transferase | 157.0387156 | 50.14184762 | 0.011778547 |
| EC042_2150 | CBG34976.1 | putative phage terminase large subunit | 140.8063486 | 52.05546208 | 0.013423464 |
| EC042_0584 | CBG33411.1 | hypothetical protein | 6.013398362 | 0 | 0.013505881 |
| EC042_3045 | CBG35872.1 | putative transcriptional regulator | 31.67711931 | 67.71872896 | 0.014984778 |
| EC042_3214 | CBG36042.1 | conserved hypothetical protein | 48.13995991 | 91.88909494 | 0.015359443 |
| EC042_1629 | CBG34451.1 | putative arylsulfatase-activating protein | 18.82887233 | 41.16041676 | 0.01731902 |
| EC042_3493 | CBG36319.1 | probable sigma(54) modulation protein | 61.90903865 | 142.5211835 | 0.017969874 |
| EC042_4576 | CBG37397.1 | putative type VI secretion protein | 2555.75515 | 414.9080134 | 0.019767557 |
| EC042_3801 | CBG36626.1 | hemin ABC transporter, ATP-binding protein | 62.84519445 | 124.6549114 | 0.019891767 |
| EC042_2148 | CBG34974.1 | putative phage prohead protease | 124.9847067 | 54.35505691 | 0.01995843 |
| EC042_4003 | CBG36829.1 | sugar efflux transporter C | 52.59239029 | 91.38256663 | 0.020005252 |
| EC042_4080 | CBG36905.1 | phosphate ABC transporter, substrate-binding protein | 44.44343376 | 88.65674573 | 0.020043565 |
| EC042_4623 | CBG37449.1 | elongation factor P | 92.66638866 | 209.0980696 | 0.020520985 |
| EC042_3205 | CBG36033.1 | conserved hypothetical protein | 27.65180056 | 56.77538881 | 0.022332502 |
| EC042_4454 | CBG37276.1 | multidrug resistance protein | 41.45365232 | 92.33141928 | 0.022632741 |
| EC042_3554 | CBG36379.1 | ABC transporter, ATP-binding protein | 208.8598776 | 508.0669809 | 0.023150933 |
| EC042_3970 | CBG36795.1 | putative prophage protein | 9.23365336 | 24.49195325 | 0.023661269 |
| EC042_1020 | CBG33843.1 | outer membrane protein F, OmpF | 180.6334869 | 71.94017652 | 0.023832125 |
| EC042_4474 | CBG37296.1 | conserved hypothetical protein | 27.25746194 | 55.6818408 | 0.023889919 |
| EC042_0204 | CBG33036.1 | membrane-bound lytic murein transglycosylase D precursor | 214.2822993 | 538.9513794 | 0.024054525 |
| EC042_4084 | CBG36909.1 | putative major fimbrial subunit, lpfA | 25.69649396 | 49.03220133 | 0.0243471 |
| EC042_4466 | CBG37288.1 | phosphonate metabolism protein | 16.77524669 | 40.58949534 | 0.024460761 |
| EC042_4456 | CBG37278.1 | metallo-beta-lactamase superfamily protein | 115.9804644 | 217.3161562 | 0.025350161 |
| EC042_4582 | CBG37407.1 | hypothetical protein | 369.5459599 | 128.2170862 | 0.026141868 |
| EC042_0340 | CBG33174.1 | conserved hypothetical protein | 19.19043794 | 44.87514687 | 0.02737407 |
| EC042_4288 | CBG37113.1 | putative extracytoplasmic stress resistance protein, CpxP | 102.4756947 | 45.41387174 | 0.027570219 |
| EC042_3204 | CBG36032.1 | putative regulatory protein | 12.86463349 | 28.1022337 | 0.027610447 |
| EC042_3196 | CBG36020.1 | probable microcin H47 secretion/processing ATP-binding protein | 80.21141784 | 136.5873499 | 0.02807868 |
| EC042_0084 | CBG32918.1 | cell division protein FtsL | 70.83134839 | 170.6475646 | 0.028102901 |
| EC042_1292 | CBG34112.1 | DNA-binding protein (histone-like protein Hlp-II), H-NS | 228.5419108 | 486.0195069 | 0.028617266 |
| EC042_4209 | CBG37036.1 | 5-methyltetrahydropteroyltriglutamate-homocysteine methyltransferase | 834.3643759 | 283.201573 | 0.029262936 |
| EC042_0705 | CBG33528.1 | N-acetylglucosamine-6-phosphate deacetylase | 122.5541914 | 60.90024672 | 0.029897949 |
| EC042_3237 | CBG36066.1 | putative capsular polysaccharide modification protein | 866.8216727 | 260.1728607 | 0.030117162 |
| EC042_2158 | CBG34984.1 | putative prophage protein | 75.21717028 | 144.9576137 | 0.030377065 |
| EC042_4075 | CBG36900.1 | putative type III effector protein | 48.94502366 | 77.97883801 | 0.030573687 |
| EC042_3229 | CBG36057.1 | conserved hypothetical protein | 31.95675238 | 66.18304574 | 0.031442276 |
| EC042_2823 | CBG35656.1 | hypothetical protein | 207.1012091 | 434.8887501 | 0.031751708 |
| EC042_3144 | CBG35969.1 | transketolase 1 | 327.4553588 | 1031.299317 | 0.031893694 |
| EC042_1635 | CBG34457.1 | fimbrial protein, fimA | 20.45538633 | 40.38845601 | 0.032195344 |
| EC042_3335 | CBG36163.1 | fimbrial protein | 16.8080197 | 36.97116575 | 0.032692047 |
| EC042_3655 | CBG36480.1 | putative fimbrial assembly protein, PilN | 78.0779846 | 41.6508468 | 0.033180662 |
| EC042_3928 | CBG36751.1 | putative lipopolysaccharide biosynthesis protein | 115.1590141 | 222.7754689 | 0.034833471 |
| EC042_4753 | CBG37572.1 | conserved hypothetical protein | 114.2054094 | 204.5230273 | 0.035378764 |
| EC042_3610 | CBG36435.1 | FKBP-type peptidyl-prolyl cis-trans isomerase | 78.35549274 | 155.6757671 | 0.037011568 |
| EC042_1487 | CBG34311.1 | phage lysozome | 12.83186047 | 29.35657534 | 0.037770078 |
| EC042_0902 | CBG33727.1 | DNA protection during starvation protein,dps | 916.2900021 | 280.5075704 | 0.038446188 |
| EC042_4461 | CBG37283.1 | ribose 1,5-bisphosphokinase | 9.578832462 | 25.60964868 | 0.039210624 |
| EC042_4463 | CBG37285.1 | phosphonates transport ATP-binding protein | 27.22468893 | 53.52694133 | 0.039385824 |
| EC042_4712 | CBG37532.1 | conserved hypothetical protein | 62.71516487 | 31.90550433 | 0.039802025 |
| EC042_4469 | CBG37291.1 | GntR-family transcriptional regulator | 11.20534647 | 25.44885504 | 0.040999009 |
| EC042_3690 | CBG36514.1 | fimbrial protein, aaspE | 7.212800733 | 17.52072651 | 0.041786101 |
| EC042_4470 | CBG37292.1 | phosphonate ABC transporter, permease protein | 37.24701953 | 64.93675323 | 0.042688262 |
| EC042_1831 | CBG34658.1 | riboflavin synthase alpha chain | 63.48639314 | 127.8228675 | 0.042760732 |
| EC042_4143 | CBG36969.1 | LysR-family transcriptional regulator (H-NS-dependent flhD  regulator) | 78.2407872 | 159.2940966 | 0.043404176 |
| EC042_2223 | CBG35049.1 | AMP nucleosidase | 79.7679197 | 129.3989856 | 0.043635433 |
| EC042_4499 | CBG37321.1 | transcriptional activator,CadC | 81.00009508 | 126.0380393 | 0.043962359 |
| EC042_0445 | CBG33278.1 | putative endonuclease | 45.98801523 | 88.76924454 | 0.04429536 |
| EC042_0592 | CBG33419.1 | hypothetical protein | 2.776756857 | 0 | 0.044714732 |
| EC042_3773 | CBG36597.1 | conserved hypothetical protein | 30.42855742 | 51.1389951 | 0.045111 |
| EC042_3822 | CBG36647.1 | putative lipoprotein | 6.013398362 | 14.99594494 | 0.045135695 |
| EC042_4514 | CBG37337.1 | conserved hypothetical protein | 9.578832462 | 25.77044231 | 0.045440576 |
| EC042_0828 | CBG33654.1 | hypothetical protein | 162.1487312 | 301.0193611 | 0.046193733 |
| EC042_4459 | CBG37281.1 | carbon-phosphorus lyase complex accessory protein (metallo-beta-lactamase superfamily protein) | 13.17703958 | 30.49036905 | 0.046314295 |
| EC042_4555 | CBG37377.1 | putative transcriptional regulator, PerC | 4.797609484 | 16.37888366 | 0.046789189 |
| EC042_2226 | CBG35052.1 | LysR-family transcriptional regulator | 98.81087908 | 177.2249509 | 0.046998744 |
| EC042_1634 | CBG34456.1 | fimbrial adhesin | 22.45985245 | 38.8125271 | 0.047017885 |
| EC042_2206 | CBG35032.1 | conserved hypothetical protein | 82.82430963 | 146.9758669 | 0.04719755 |
| EC042_3118 | CBG35943.1 | 2-octaprenyl-6-methoxyphenol hydroxylase | 100.7846971 | 53.96121653 | 0.048023617 |
| EC042_4083 | CBG36908.1 | fimbrial chaperone protein, LpfB | 32.10423094 | 53.36614769 | 0.048990001 |
| EC042_3931 | CBG36754.1 | lipopolysaccharide heptosyltransferase 1 | 82.01924588 | 148.8814322 | 0.049237527 |
| EC042_0588 | CBG33415.1 | hypothetical protein | 4.008932241 | 0 | 0.049280655 |
| EC042_3208 | CBG36036.1 | hypothetical protein | 16.05211547 | 30.12048695 | 0.049512221 |
| EC042_4009 | CBG36835.1 | putative type III effector protein | 51.75455353 | 77.76170041 | 0.049823377 |
