# Supplementary material for: The AraC Negative Regulator family modulates the activity of histone-like proteins in pathogenic bacteria
Source: PLoS Pathog. 2017 Aug 14;13(8):e1006545. doi: 10.1371/journal.ppat.1006545 (PMC5570504; doi:10.1371/journal.ppat.1006545)
Supplement: S5 Table — (DOCX) [file ppat.1006545.s016.docx]

| **pAA genes** | | **RNA-seq**  **042 vs 042*aar***  **Product** | **042** | **042*aar*** |  |
| --- | --- | --- | --- | --- | --- |
| **ORF** | **Protein Id** |  | **Read.Count.G1** | **Read.Count.G2** | **p.value** |
| EC042_pAA060 | CBG27812.1 | conserved hypothetical protein, Aar | 25.0255711 | 0.640200079 | 2.15E-06 |
| EC042_pAA048 | CBG27800.1 | major fimbrial subunit of aggregative adherence fimbria II, AafA | 3290.927711 | 8426.896456 | 6.80E-05 |
| EC042_pAA008 | CBG27762.1 | outer membrane protein, AatA | 70.95998863 | 203.2862068 | 0.00195695 |
| EC042_pAA030 | CBG27784.1 | afimbrial adhesion, AafB | 176.5318759 | 429.2168825 | 0.01045538 |
| EC042_pAA047 | CBG27799.1 | hypothetical protein | 316.6148396 | 866.7803788 | 0.00996196 |
| EC042_pAA005 | CBG27758.1 | hypothetical protein | 55.83375907 | 125.3260339 | 0.01272778 |
| EC042_pAA046 | CBG27798.1 | chaperone protein, AafD | 343.2298992 | 882.9387221 | 0.0171349 |
| EC042_pAA061 | CBG27813.1 | conserved hypothetical protein | 93.02876233 | 209.4297103 | 0.01979857 |
| EC042_pAA005A | CBG27759.1 | conserved hypothetical protein | 72.17449003 | 143.9699977 | 0.02711053 |
| EC042_pAA086 | CBG27838.1 | conjugative transfer protein, TraW | 56.21836663 | 118.5933376 | 0.03070946 |
| EC042_pAA108 | CBG27860.1 | conserved hypothetical protein | 66.04611463 | 30.96706989 | 0.05025372 |
| EC042_pAA052 | CBG27804.1 | transcriptional activator, AggR | 320.1941491 | 659.2922999 | 0.05286497 |
