# Supplementary material for: The AraC Negative Regulator family modulates the activity of histone-like proteins in pathogenic bacteria
Source: PLoS Pathog. 2017 Aug 14;13(8):e1006545. doi: 10.1371/journal.ppat.1006545 (PMC5570504; doi:10.1371/journal.ppat.1006545)
Supplement: S6 Table — (DOCX) [file ppat.1006545.s017.docx]

| pAA plasmid | | RNA-seq  042*aar*(pAar) vs 042*aar*  Product | 042*aar*(pAar) | 042*aar* |  |
| --- | --- | --- | --- | --- | --- |
| Feature.ID | Protein Id |  | Read.Count.G3 | Read.Count.G2 | p.Value |
| EC042_pAA048 | CBG27800.1 | major fimbrial subunit of aggregative adherence fimbria II, AafA | 142.3109781 | 8113.81064 | 1.98E-20 |
| EC042_pAA060 | CBG27812.1 | conserved hypothetical protein, Aar | 713.2878727 | 0.579319849 | 1.20E-16 |
| EC042_pAA005A | CBG27759.1 | conserved hypothetical protein | 6.488041289 | 140.2124019 | 2.86E-10 |
| EC042_pAA047 | CBG27799.1 | hypothetical protein | 15.41670396 | 831.0584745 | 6.68E-09 |
| EC042_pAA055 | CBG27807.1 | Dispersin, Aap | 44.33156841 | 1058.127435 | 2.91E-06 |
| EC042_pAA005 | CBG27758.1 | hypothetical protein | 16.9466307 | 122.1074446 | 1.62E-05 |
| EC042_pAA003 | CBG27756.1 | hypothetical protein | 48.06724338 | 422.1074414 | 2.18E-05 |
| EC042_pAA030 | CBG27784.1 | afimbrial adhesion, AafB | 53.66061463 | 413.0792931 | 2.11E-05 |
| EC042_pAA046 | CBG27798.1 | chaperone protein, AafD | 71.00011984 | 850.1224609 | 8.52E-05 |
| EC042_pAA052 | CBG27804.1 | transcriptional activator, AggR | 77.0995444 | 632.196028 | 0.0002816 |
| EC042_pAA008 | CBG27762.1 | outer membrane protein, AatA | 62.4963809 | 195.43034 | 0.00883591 |
| EC042_pAA127 | CBG27876.1 | conserved hypothetical protein | 71.88201652 | 22.18210256 | 0.01125628 |
| EC042_pAA145 | CBG27893.1 | major facilitator superfamily protein, EitD | 139.1422036 | 60.15188305 | 0.03144393 |
| EC042_pAA108 | CBG27860.1 | conserved hypothetical protein | 69.44565292 | 29.7497711 | 0.04466888 |
